# Periostin‐CCL3 Feedforward Signaling Loop Promotes Cardiac Fibrosis and Cardiomyocyte Necroptosis in Arrhythmogenic Cardiomyopathy

**DOI:** 10.1002/advs.77670

**Published:** 2026-09-10

**Authors:** Tiantian Wu, Ruotong Li, Xiaoyue Zhang, Dandan Chen, Jianmeng Yang, Mengmeng Li, Yuhan Yin, Ya Wang, Yuqiu Tian, Haoling Yan, Dan Cui, Jiangping Song, Gaoliang Ouyang

**Affiliations:** ^1^ Institute For Common Diseases JinJiang Municipal Hospital School of Medicine School of Life Sciences Xiamen University Xiamen China; ^2^ State Key Laboratory of Cellular Stress Biology School of Life Sciences State Key Laboratory of Vaccines for Infectious Diseases Xiang An Biomedicine Laboratory Xiamen University Xiamen China; ^3^ State Key Laboratory of Cardiovascular Disease Fuwai Hospital Chinese Academy of Medical Sciences and Peking Union Medical College Beijing China; ^4^ Guangzhou National Laboratory Guangzhou China

**Keywords:** arrhythmogenic cardiomyopathy, cardiac fibrosis, cardiomyocyte necroptosis, CCL3, periostin

## Abstract

Periostin (POSTN) is a multifunctional extracellular matrix protein in cardiovascular diseases. However, its role in arrhythmogenic cardiomyopathy (ACM) remains unknown. Here, we demonstrate that POSTN is significantly elevated in the myocardium of ACM mice and patients. POSTN deficiency alleviates heart dysfunction, reduces cardiac fibrosis and improves survival rates in ACM mice. POSTN is mainly derived from cardiac myofibroblasts and promotes cardiomyocyte necroptosis in vivo and in vitro. Mechanistically, POSTN facilitates cardiomyocyte necroptosis through the integrin‐JNK‐RIP3‐MLKL signaling and induces CCL3 expression via the integrin‐JNK‐ETS2 pathway. In turn, CCL3 activates cardiac myofibroblasts and stimulates POSTN expression through the NF‐κB signaling. Our findings show that POSTN and CCL3 facilitate ACM progression through the POSTN‐integrin‐JNK‐ETS2‐CCL3 signaling in cardiomyocytes and CCL3‐CCR5‐NF‐κB‐POSTN axis in cardiac myofibroblasts. We further found that pharmacological inhibition of either JNK or CCR5 to target JNK‐CCL3‐CCR5 axis attenuates subsequent ACM progression in mice by suppressing cardiac fibrosis and cardiomyocyte necroptosis. Therefore, targeting the POSTN‐CCL3 feedforward signaling loop may offer a novel therapeutic strategy for patients with ACM.

## Graphical abstract

POSTN‐CCL3 signaling forms a feed‐forward circuit between cardiomyocytes and cardiac myofibroblasts in arrhythmogenic cardiomyopathy. POSTN activates JNK/RIP3‐dependent necroptotic signaling and JNK/ETS2‐induced CCL3 expression in cardiomyocytes. In turn, CCL3‐CCR5 signaling in cardiac myofibroblasts activates NF‐κB/p65 and promotes POSTN expression, thereby sustaining myocardial injury and fibrosis and contributing to ACM progression.

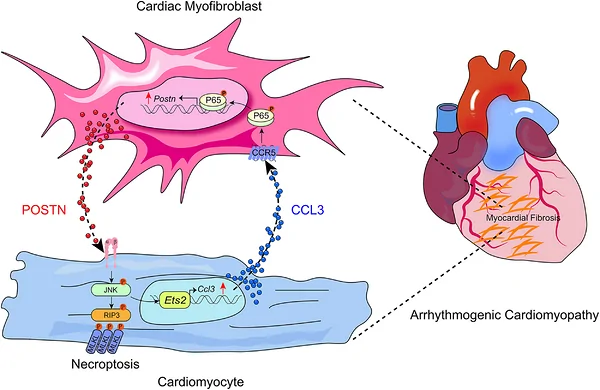

## 1 Introduction

Arrhythmogenic cardiomyopathy (ACM) is an inherited myocardial disorder characterized by arrhythmias and structural defects of the myocardium. ACM remains a major cause of sudden cardiac death among familial genetic cardiomyopathies [1, 2]. ACM results in the loss of cardiomyocytes, which are replaced by fibrofatty tissue. Approximately 50% to 60% of ACM patients have been associated with genetic mutations in desmosomal genes, including *DSP*, *PKP2*, *JUP*, *DSC2*, and *DSG2* [3, 4, 5]. Desmosomes are key regulators for preserving the structural stability of cardiomyocytes and enhancing cardiac resilience during contractile cycles by facilitating intercellular adhesion. Mutations in desmosomal genes disrupt intercellular communication, thereby accelerating disease progression [6, 7]. Desmoplakin (DSP), a critical structural protein, connects desmosomes to the cytoskeletal network. Mutations in *DSP* result in a distinct cardiomyopathy variant characterized by increased cardiac fibrosis, severe arrhythmias, and intermittent myocardial inflammation, leading to ventricular dysfunction, enlargement, and further myocardial injury [8, 9].

Periostin (encoded by *Postn*) is a multifunctional extracellular matrix (ECM) protein that plays important roles in tissue repair, inflammation, pathological fibrosis, and tumor progression [10, 11, 12]. Recent studies have highlighted POSTN's regulatory role in various cardiovascular diseases, including pulmonary hypertension [13], myocardial infarction [14, 15], cardiac fibrosis [16], and heart failure [17]. However, its role in ACM remains poorly understood. POSTN expression is low in the healthy adult heart, but it is significantly increased in damaged cardiac tissue and injured vascular walls [18], particularly in vascular smooth muscle cells and cardiac fibroblasts [19, 20, 21]. Shimazaki et al. found that POSTN recruits activated fibroblasts by FAK‐integrin signaling, contributing to myocardial healing after acute myocardial infarction. As a regulator of cardiac hypertrophy and ventricular remodeling, POSTN, induced by myocardial infarction‐activated cardiac fibroblasts, leads to collagenous scar formation [18]. Persistent interstitial fibrosis with increased POSTN expression results in ventricular wall thinning, progressive chamber dilation, and impaired cardiac function [22, 23]. POSTN is upregulated in heart failure induced by diabetic cardiomyopathy, while treatment with resveratrol reduces fibroblast activation and inhibits POSTN secretion through ERK/TGF‐β signaling [24]. However, the role and mechanism of POSTN in ACM remains unknown.

In this study, we used the *DSP* conditionally knocking‐down ACM mice to identify the role of POSTN in ACM. We found that POSTN is markedly upregulated in ACM myocardium and POSTN deficiency alleviates cardiac dysfunction, reduces cardiac fibrosis, and improves survival rates in ACM mice. Our findings reveal that POSTN promotes ACM through the integrin‐JNK‐RIP3‐MLKL signaling‐mediated cardiomyocyte necroptosis. Furthermore, POSTN‐dependent *Ets2* activation in cardiomyocytes induces CCL3 expression. Subsequently, CCL3 activates NF‐κB signaling and POSTN expression in cardiac fibroblasts, creating a vicious cycle between cardiomyocytes and fibroblasts. POSTN‐CCL3 feedforward signaling loop may amplify the pathological interplay between cardiomyocytes and myofibroblasts, leading to cardiomyocyte necroptosis, fibrosis, and contributing to ACM progression. Importantly, POSTN is positively correlated with cardiomyocyte necroptosis and CCL3 upregulation in myocardial tissues of patients with ACM. Pharmacological inhibition of either JNK or CCR5 to target JNK‐CCL3‐CCR5 axis attenuates ACM progression in mice by suppressing cardiac fibrosis and cardiomyocyte necroptosis. These findings suggest that targeting POSTN‐CCL3 signaling loop may represent potential therapeutic strategies for the treatment of ACM.

## 2 Results

### 2.1 POSTN is Highly Increased in the Myocardium of ACM Mice

**FIGURE 1:**
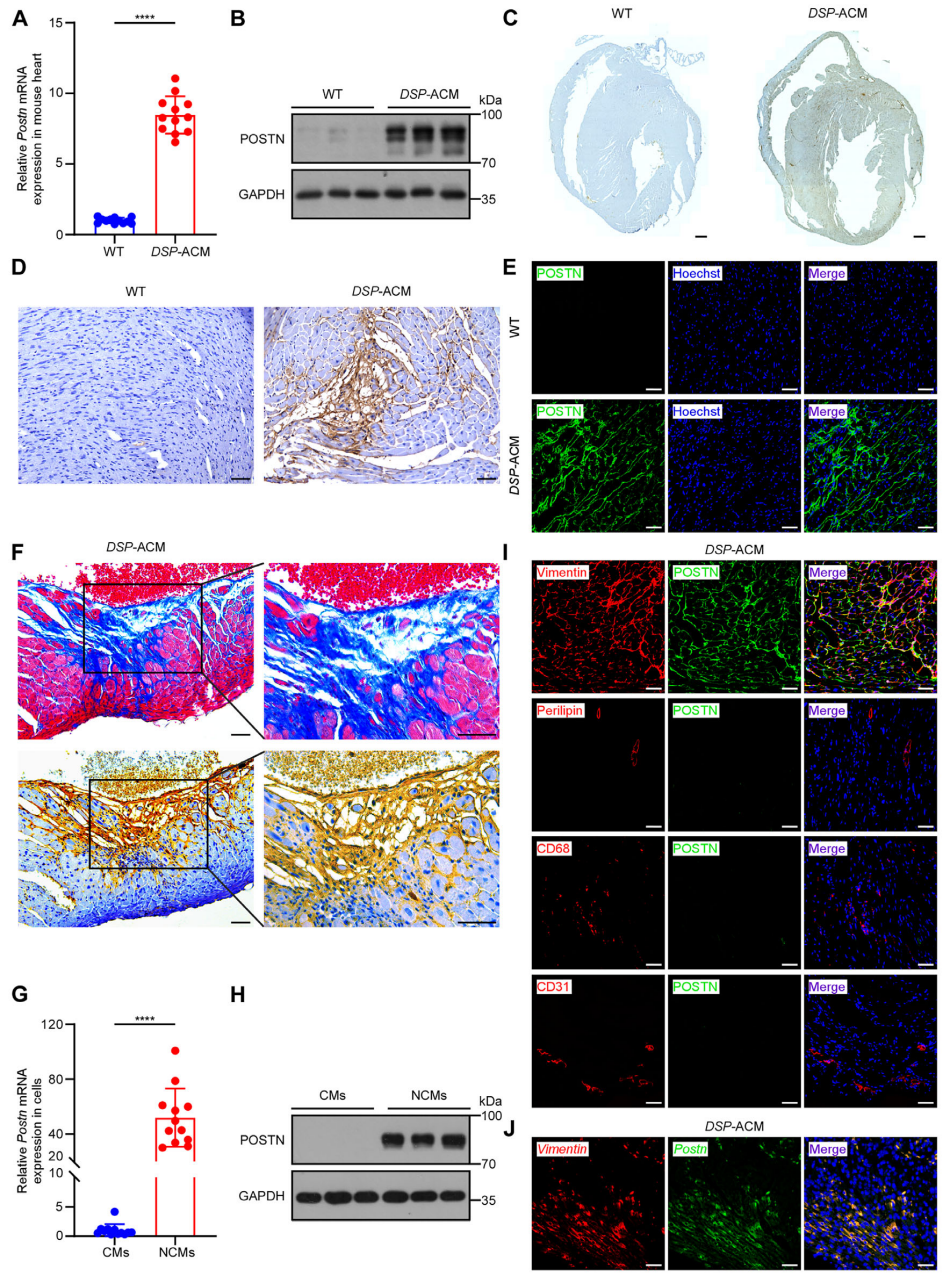
POSTN is upregulated in the myocardium of *DSP*‐ACM mice and predominantly derived from cardiac fibroblasts. (A,B) The mRNA (A) (*n* = 12) and protein (B) levels of POSTN in the myocardium of WT (*DSP^f/+^*) and *DSP*‐ACM (*CreDSP^f/+^*) mice. (C–E) Representative images of immunohistochemical (C,D) and immunofluorescence (E) staining of POSTN from WT and *DSP*‐ACM mouse cardiac sections. Scale bars, 500 µm (C) and 50 µm (D,E). (F) Masson's Trichrome staining and immunohistochemical staining of POSTN in cardiac continuous sections from *DSP*‐ACM mice. Scale bars, 50 µm. (G,H) The mRNA (G) (*n* = 12) and protein (H) levels of POSTN in CMs and NCMs isolated from the hearts of *DSP*‐ACM mice. (I) Representative immunofluorescence co‐staining of POSTN with cardiac myofibroblasts (Vimentin), adipocytes (Perilipin), macrophage (CD68), and endothelial cells (CD31) in cardiac sections of *DSP*‐ACM mice. Scale bars, 50 µm. (J) RNA‐FISH staining of cardiac myofibroblasts (*Vimentin*) and *Postn* in cardiac sections of *DSP*‐ACM mice. Scale bars, 50 µm. Statistical analyses were performed by unpaired two‐tailed Student's *t*‐test (A,G). Data are mean ± SEM. ^****^ *p* < 0.0001.

To investigate whether POSTN is related to ACM progression, we applied an ACM mouse model which *DSP* in the myocardium was conditionally knocked down [25]. POSTN expression was markedly elevated during the progression of ACM, particularly in the cardiac marginal disease zone (Figure S1A). The mRNA and protein levels of POSTN were significantly increased in the myocardium of ACM mice relative to WT littermates (Figure 1A,B). Immunohistochemical and immunofluorescence staining also showed upregulated POSTN levels in the myocardium of ACM mice (Figure 1C–E). Additionally, the levels of POSTN in the serum of ACM mice were significantly elevated (Figure S1B). Collectively, these findings demonstrate that POSTN is highly increased in the myocardium and serum of ACM mice.

In ACM mice, POSTN predominantly accumulated in the fibrotic regions of the heart, as confirmed by continuous sections with Masson's Trichrome staining and immunohistochemical staining (Figure 1F). To identify the cellular source of POSTN in the hearts of ACM mice, we isolated cardiomyocytes (CMs) and non‐cardiomyocytes (NCMs) [26]. POSTN is mainly derived from NCMs rather than CMs, as determined by qRT‐PCR and Western blot assays (Figure 1G,H). Immunofluorescence co‐staining confirmed that POSTN co‐localized with the fibroblast marker vimentin, rather than with the adipocyte marker perilipin, macrophage marker CD68, or endothelial cell marker CD31 in the myocardium of ACM mice (Figure 1I). RNA‐fluorescence in situ hybridization (RNA‐FISH) further confirmed that *Postn* mRNA co‐localized with *vimentin* in the myocardium of ACM mice (Figure 1J). Isolated fibroblasts from ACM mice exhibited elevated mRNA and protein levels of POSTN compared to those from WT mice (Figure S1C,D). These findings indicate that POSTN is predominantly derived from cardiac fibroblasts (CFs) in ACM mice. Interestingly, we found more vimentin^+^ fibroblasts in the CFs isolated from ACM mice compared to WT mice (Figure S1E). Moreover, treatment with recombinant mouse POSTN protein (rmPOSTN) increased α‐SMA expression and vimentin^+^ myofibroblasts compared to PBS treatment, suggesting that POSTN can activate myocardial fibroblasts (Figure S1F–H). Collectively, these data demonstrate that POSTN primarily originates from CFs in ACM mice.

### 2.2 POSTN Deficiency Alleviates Cardiac Dysfunction and Improves Survival in ACM Mice

**FIGURE 2:**
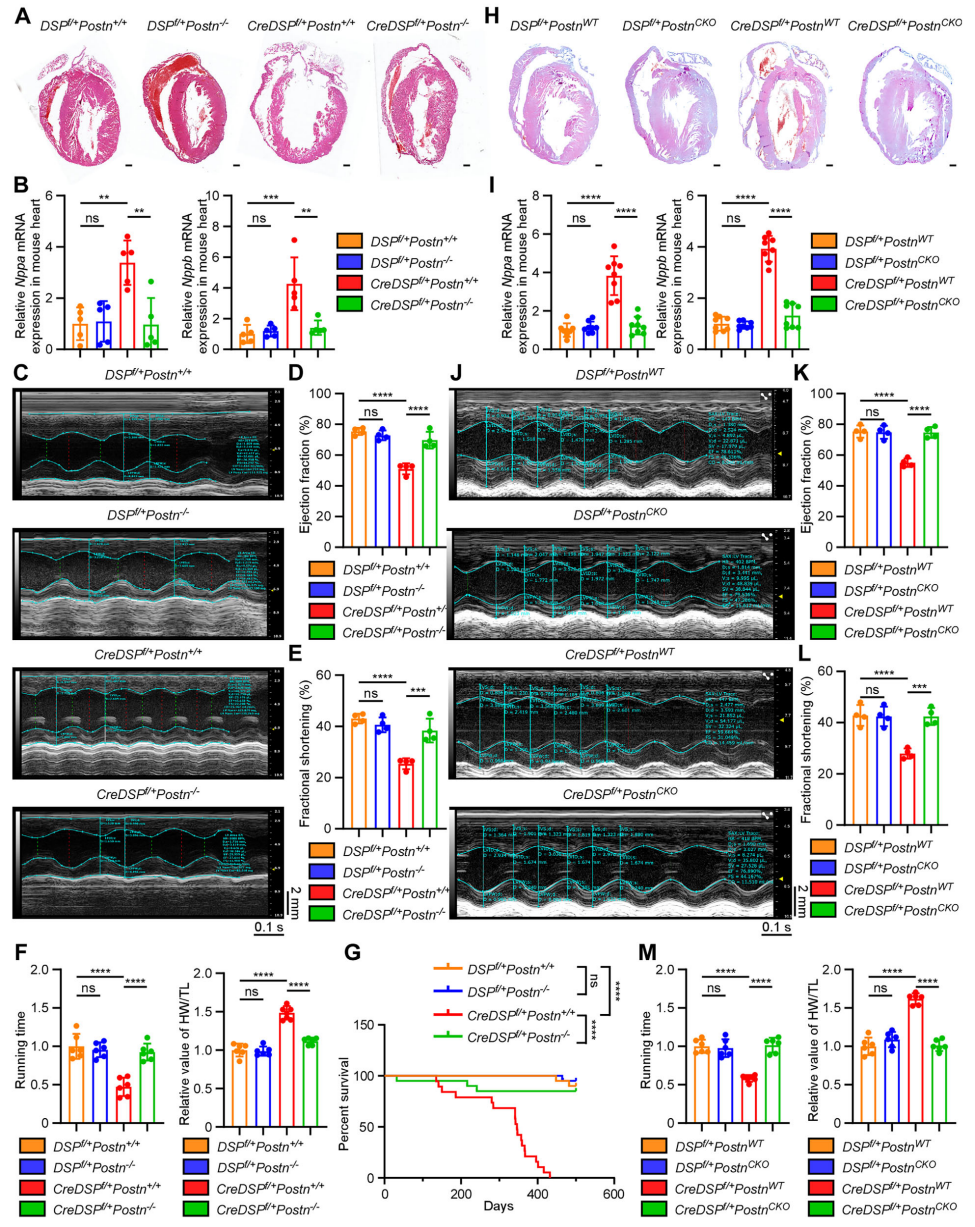
POSTN deficiency attenuates cardiac dysfunction and increases the survival rate of *DSP*‐ACM mice. (A) H&E staining in cardiac sections from WT and *DSP*‐ACM mice with or without POSTN deficiency. Scale bars, 500 µm. (B) mRNA levels of *Nppa* and *Nppb* in the myocardium of WT and *DSP*‐ACM mice with or without POSTN deficiency (n = 5). (C–E) Representative images of echocardiographs (C) and statistical data (D,E) on EF%, FS% in each group (*n* = 4). Scale bars, 2 mm. (F) Running time and HW/TL ratio in WT and *DSP*‐ACM mice with or without POSTN deficiency (*n* = 6). (G) Kaplan‐Meier survival curve analysis of WT and *DSP*‐ACM mice with or without POSTN deficiency (*n* = 20). (H) H&E staining in cardiac sections from WT and *DSP*‐ACM mice with or without AAV‐mediated cardiac fibroblast‐targeted deletion of *Postn*. Scale bars, 500 µm. (I) mRNA levels of *Nppa* and *Nppb* in the myocardium of WT and *DSP*‐ACM mice with or without AAV‐mediated cardiac fibroblast‐targeted deletion of *Postn* (*n* = 8). (J–L) Representative images of echocardiographs (J) and statistical data (K,L) on EF%, FS% in each group (*n* = 4). Scale bars, 2 mm. (M) Running time and HW/TL ratio in WT and *DSP*‐ACM mice with or without AAV‐mediated cardiac fibroblast‐targeted deletion of *Postn* (*n* = 6). Statistical analyses were performed by one‐way ANOVA analysis followed by Tukey's multiple comparison tests (B,D–F,I,K–M). Survival curves are compared using the log‐rank test (G). Data are mean±SEM. ^**^ *p* < 0.01, ^***^ *p* < 0.001, ^****^ *p* < 0.0001. ns, no significance.

Ventricular involvement induced by ACM, rendered the ventricular walls more susceptible to wall stress, resulting in cardiac function deterioration. To explore the effect of POSTN on cardiac dysfunction in ACM mice, we employed hematoxylin and eosin (H&E) staining and panoramic scanning to detect cardiac morphological alterations. The ventricular wall of *CreDSP^f/+^Postn^+/+^* mice displayed progressive thinning and abnormal shaping compared to *CreDSP^f/+^Postn^−/−^* mice. Histological analysis revealed partial loss of myocardium in *CreDSP^f/+^Postn^+/+^* mice (Figure 2A and Figure S2A). The expression of cardiac failure‐associated genes, including *Nppa* and *Nppb*, were remarkably decreased in the heart of *CreDSP^f/+^Postn^−/−^* mice compared to *CreDSP^f/+^Postn^+/+^* mice (Figure 2B). Electrocardiogram (ECG) detection showed ACM‐associated T‐wave inversion in *CreDSP^f/+^Postn^+/+^* mice, which was absent in *CreDSP^f/+^Postn^−/−^* mice (Figure S2B). Moreover, ventricular dilatation, coupled with decreased ejection fraction (EF%) and fractional shortening (FS%), was largely improved by POSTN deficiency in *CreDSP^f/+^Postn^−/−^* mice compared with *CreDSP^f/+^Postn^+/+^* mice (Figure 2C–E and Figure S2C). Importantly, although *CreDSP^f/+^Postn^−/−^* mice exhibited improved cardiac function and ECG patterns, transmission electron microscopy (TEM) analysis revealed that the ultrastructural defect of the desmosomes is not fully restored (Figure S2D). Mouse treadmill training demonstrated a significant increase in running time for *CreDSP^f/+^Postn^−/−^* mice compared to *CreDSP^f/+^Postn^+/+^* mice and the heart weight/tibial length (HW/TL) ratio was significantly reduced in *CreDSP^f/+^Postn^−/−^* mice (Figure 2F). Additionally, long‐term mortality tracking revealed a significant improvement in survival rates in *CreDSP^f/+^Postn^−/−^* mice compared to *CreDSP^f/+^Postn^+/+^* mice, indicating that POSTN deficiency enhances the survival of ACM mice (Figure 2G).

Importantly, AAV9‐*Tcf21*‐Cre‐mediated cardiac fibroblast‐targeted deletion of *Postn* in ACM mice recapitulated the protective effects of global *Postn* deficiency, including reduced myocardial injury, decreased expression of *Nppa* and *Nppb*, improved cardiac function, and a lower HW/TL ratio (Figure 2H–M and Figure S2E–H).

Inflammatory infiltration can be detected in over two‐thirds of hearts affected by ACM, particularly in zones of fibrofatty replacement, correlating with more severe biventricular involvement [27]. Considering the heightened myocardial inflammation during ACM progression, we detected whether POSTN deficiency influenced inflammation in ACM mice. qRT‐PCR analysis revealed that the expression of inflammation‐related genes, such as *CD68*, *F4/80*, *Tnf‐α*, *IL‐6*, *IL‐1β*, and *IL‐17*, was significantly reduced in *CreDSP^f/+^Postn^−/−^* mice relative to *CreDSP^f/+^Postn^+/+^* mice (Figure S3A). Immunofluorescence staining also revealed that POSTN deficiency decreased the infiltration of CD68‐positive macrophages in the myocardium of ACM mice (Figure S3B). Additionally, the serum levels of the IL‐6, which had previously been discovered to be elevated in ACM patients, were significantly lower in the serum of *CreDSP^f/+^Postn^−/−^* mice compared to those in *CreDSP^f/+^Postn^+/+^* mice (Figure S3C). These results suggest that POSTN deficiency effectively reduces myocardial inflammation in ACM mice.

### 2.3 POSTN Deficiency Attenuates Cardiac Fibrosis in ACM Mice

**FIGURE 3:**
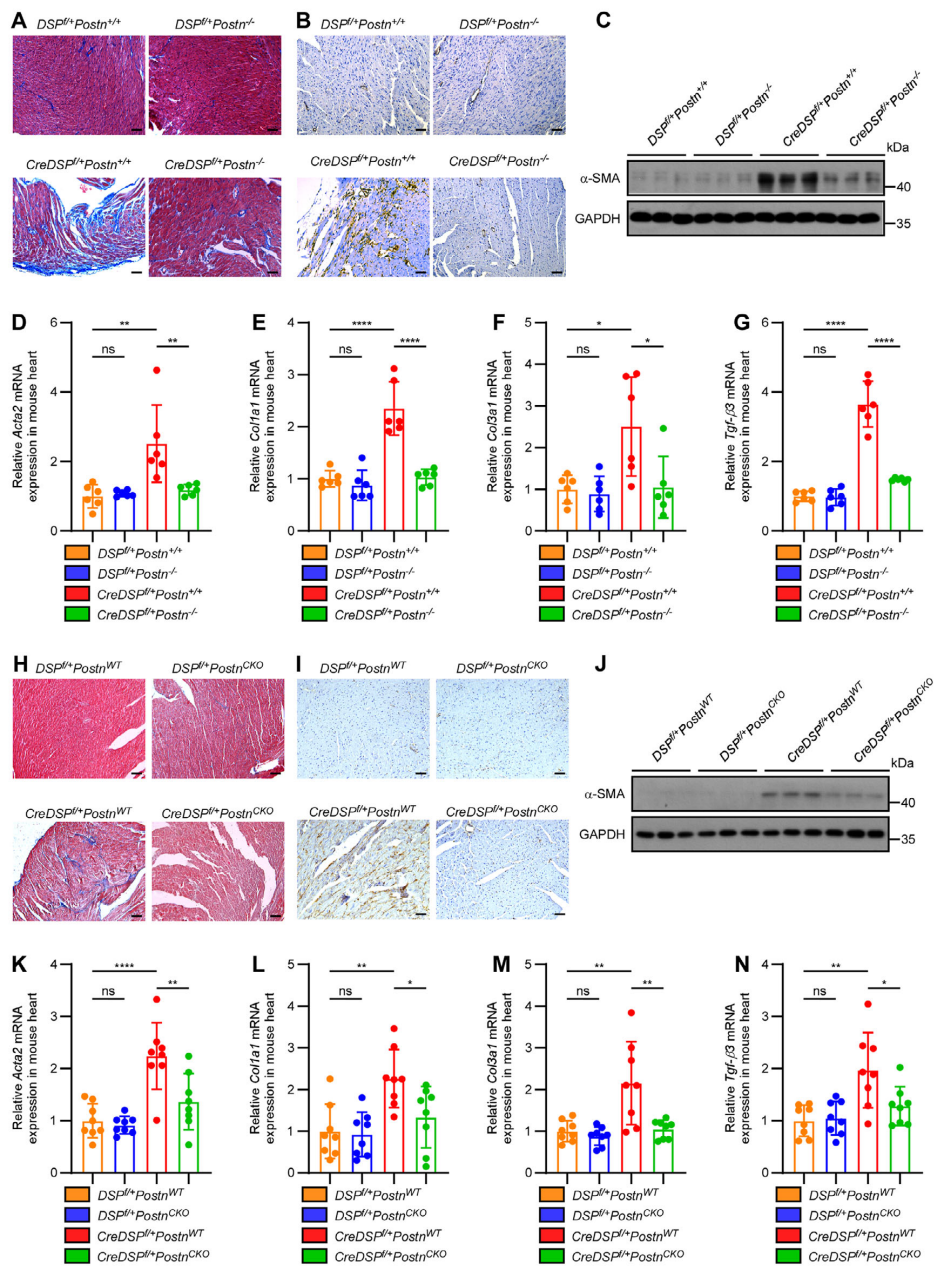
POSTN deficiency alleviates cardiac fibrosis in *DSP*‐ACM mice. (A,B) Masson's Trichrome staining (A) and immunohistochemical staining of α‐SMA (B) in cardiac sections from WT and *DSP*‐ACM mice with or without POSTN deficiency. Scale bars, 50 µm. (C) Western blot analysis of α‐SMA in the cardiac tissue lysates of WT and *DSP*‐ACM mice with or without POSTN deficiency. (D–G) mRNA levels of *Acta2* (D), *Col1a1* (E), *Col3a1* (F), and *Tgf‐β3* (G) in the myocardium of WT and *DSP*‐ACM mice with or without POSTN deficiency (*n* = 6). (H,I) Masson's Trichrome staining (H) and immunohistochemical staining of α‐SMA (I) in cardiac sections from WT and *DSP*‐ACM mice with or without AAV‐mediated cardiac fibroblast‐targeted deletion of *Postn*. Scale bars, 50 µm. (J) Western blot analysis of α‐SMA in the cardiac tissue lysates of WT and *DSP*‐ACM mice with or without AAV‐mediated cardiac fibroblast‐targeted deletion of *Postn*. (K–N) mRNA levels of *Acta2* (K), *Col1a1* (L), *Col3a1* (M), and *Tgf‐β3* (N) in the myocardium of WT and *DSP*‐ACM mice with or without AAV‐mediated cardiac fibroblast‐targeted deletion of *Postn* (*n* = 8). Statistical analyses were performed by one‐way ANOVA analysis followed by Tukey's multiple comparison tests (D‐G, K‐N). Data are mean±SEM. ^*^ *p* < 0.05, ^**^ *p* < 0.01, ^****^ *p* < 0.0001. ns, no significance.

Fibrosis is a hallmark of ACM. To investigate whether POSTN contributes to cardiac fibrosis in ACM mice, we utilized staining methods to evaluate cardiac fibrosis in *CreDSP^f/+^Postn^+/+^* mice and *CreDSP^f/+^Postn^−/−^* mice. Masson's Trichrome staining and Sirius Red staining showed that POSTN deficiency markedly reduced collagen deposition in the myocardium lesion areas of ACM mice (Figure 3A and Figure S4A). Immunohistochemical analysis revealed a significant reduction in α‐SMA and Col1a1 expression in the myocardium of *CreDSP^f/+^Postn^−/−^* mice compared to *CreDSP^f/+^Postn^+/+^* mice (Figure 3B and Figure S4B). Western blot further confirmed that α‐SMA levels were remarkably downregulated in the myocardium of *CreDSP^f/+^Postn^−/−^* mice compared to *CreDSP^f/+^Postn^+/+^* mice (Figure 3C). Immunofluorescence staining showed that both Vimentin and α‐SMA levels were significantly decreased in the hearts of ACM mice following *Post*n deletion (Figure S4C,D). As shown in the heatmap (Figure S4E), fibrosis‐related pathways were prominently downregulated in *CreDSP^f/+^Postn^−/−^* mice compared to *CreDSP^f/+^Postn^+/+^* mice. Moreover, POSTN deficiency significantly suppressed the transcription of fibrosis‐associated genes, such as *Acta2*, *Col1a1*, *Col3a1*, *Tgf‐β3*, *Fn1*, *Ctgf*, *Mmp2*, and *Mmp9* in the cardiac tissues of ACM mice (Figure 3D–G and Figure S4F–I). Moreover, similar results were obtained in ACM mice with AAV‐mediated cardiac fibroblast‐targeted deletion of *Postn*. Masson's Trichrome staining, immunohistochemical staining, Western blot, and qPCR analysis showed significantly reduced myocardial fibrosis in *CreDSP^f/+^Postn^CKO^* mice relative to *CreDSP^f/+^Postn^WT^* mice (Figure 3H–N). These data collectively suggest that POSTN deficiency attenuates cardiac fibrosis in ACM mice.

### 2.4 POSTN Induces Cardiomyocyte Necroptosis Through the Integrin‐JNK‐RIP3‐MLKL Axis

**FIGURE 4:**
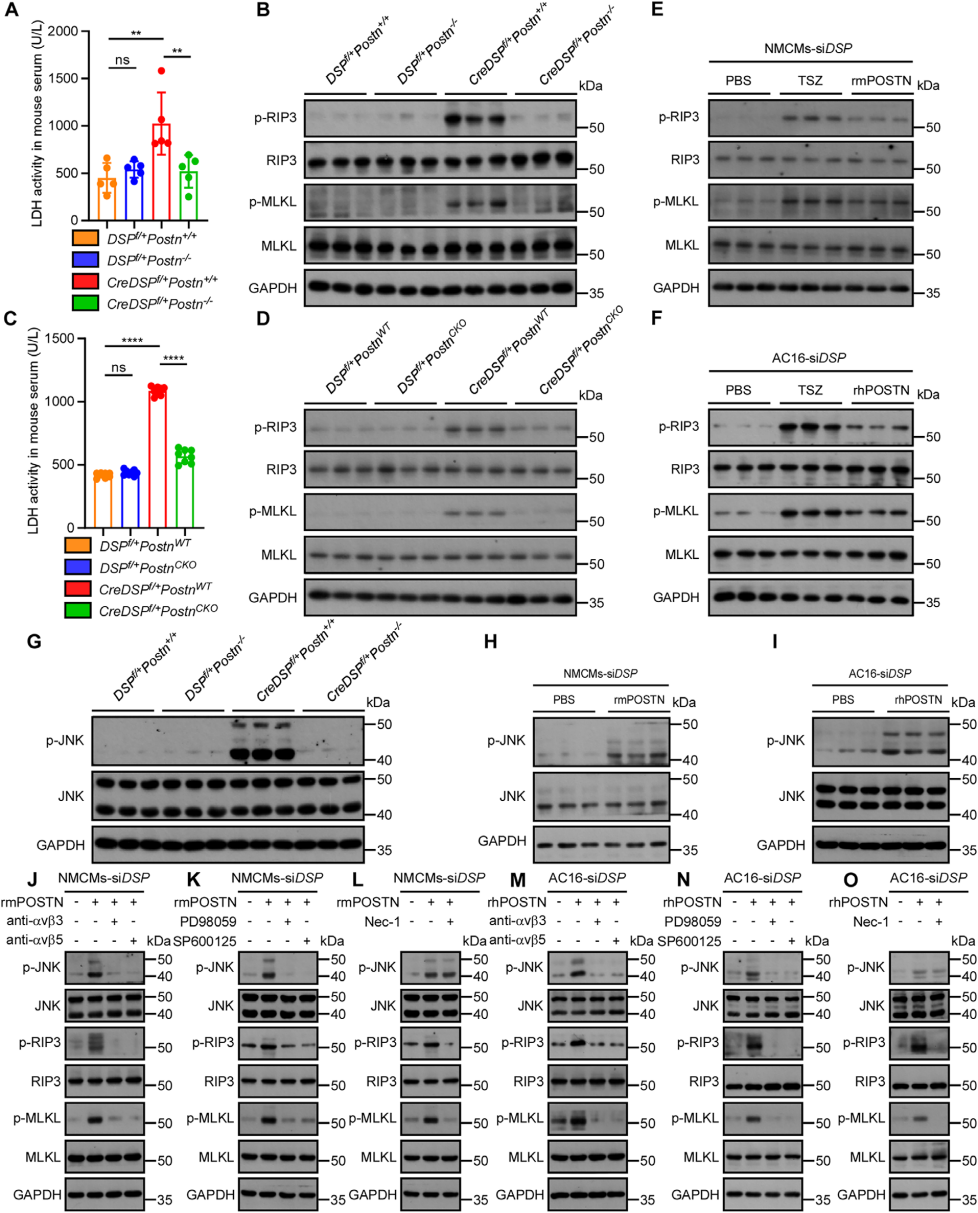
POSTN promotes cardiomyocyte necroptosis via the integrin‐JNK‐RIP3‐MLKL axis. (A,C) The activities of lactate dehydrogenase (LDH) in the serum isolated from WT and *DSP*‐ACM mice with or without either global (A) (*n* = 5) or cardiac fibroblast‐specific (C) (*n* = 8) POSTN deficiency. (B,D) Western blot analysis of the indicated proteins associated with necroptosis in the myocardium of WT and *DSP*‐ACM mice with or without either global (B) or cardiac fibroblast‐specific (D) POSTN deficiency. (E,F) Western blot analysis of the indicated proteins associated with necroptosis in NMCMs (E) and AC16 cells (F) transfected with si*DSP* in the presence or absence of TSZ, rmPOSTN or rhPOSTN. (G) Western blot analysis of p‐JNK and JNK in the myocardium of WT and *DSP*‐ACM mice with or without POSTN deficiency. (H,I) The protein levels of p‐JNK and JNK in NMCMs (H) and AC16 cells (I) transfected with si*DSP* in the presence or absence of rmPOSTN or rhPOSTN. (J–O) Western blot analysis of p‐JNK, JNK, p‐RIP3, RIP3, p‐MLKL and MLKL in NMCMs and AC16 cells transfected with si*DSP* in the presence or absence of rmPOSTN or rhPOSTN, as well as αvβ3 or αvβ5 integrin neutralizing antibody (J,M), PD98059 or SP600125 (K,N), and Nec‐1 (L,O). Statistical analyses were performed by one‐way ANOVA analysis followed by Tukey's multiple comparison tests (A,C). Data are mean±SEM. ^**^ *p* < 0.01, ^****^ *p* < 0.0001. ns, no significance.

A key pathological feature of ACM progression is the fibrofatty replacement of ventricular myocardial tissue. On the basis of revealing substantial cardiac fibrosis in the myocardium of ACM mice, we hypothesized that cardiomyocyte death might occur in ACM with POSTN contributing to this process. Our results demonstrated that POSTN deficiency significantly reduced lactate dehydrogenase (LDH) activity in the serum and decreased the levels of p‐RIP3 and p‐MLKL in the myocardium, suggesting that POSTN deficiency attenuates cardiomyocyte necroptosis in ACM mice (Figure 4A–D and Figure S5A). However, apoptosis and pyroptosis pathways were not significantly affected by POSTN deficiency in ACM mice (Figure S5B,C). Besides, LDH activity and the levels of p‐RIP3 and p‐MLKL were significantly downregulated in CMs isolated from *CreDSP^f/+^Postn^−/−^* mice compared to *CreDSP^f/+^Postn^+/+^* mice (Figure S5D,E). To further elucidate the role of POSTN in regulating cardiomyocyte necroptosis, we treated a human cardiomyocyte cell line AC16 with recombinant human POSTN protein (rhPOSTN). However, no significant changes in necroptosis markers were observed, indicating that POSTN alone is insufficient to induce cardiomyocyte necroptosis (Figure S5F). To mimic ACM conditions in vitro, small interfering RNAs (siRNAs) were employed to knock down *DSP* in neonatal mouse cardiomyocytes (NMCMs) and human cardiomyocyte cell line AC16 (Figure S5F–H). When NMCMs transfected with si*DSP* were treated with rmPOSTN, the levels of p‐RIP3 and p‐MLKL and LDH activity increased significantly, suggesting that POSTN accelerates cardiomyocyte necroptosis under ACM conditions, with TSZ treatment as a positive control (Figure 4E and Figure S5I). This enhancement of necroptosis was also observed in AC16 cells treated with si*DSP* and rhPOSTN (Figure 4F and Figure S5F,J). Consistently, the necrosis rate in AC16 cells transfected with si*DSP* following rhPOSTN treatment was elevated as determined by PI staining (Figure S5K). These data demonstrate that POSTN promotes cardiomyocyte necroptosis in ACM mice.

POSTN, as an extracellular regulator, is known to bind integrin receptors and activate downstream FAK, MAPK, and other pathways to promote cardiovascular disease progression [28]. Previous studies have demonstrated that JNK signaling, part of the MAPK family, interacts with p‐RIP3 and p‐MLKL to mediate necroptosis [29, 30]. To investigate whether POSTN regulated necroptosis through JNK signaling in ACM mice, we measured the levels of p‐JNK in the myocardium. Compared to *CreDSP^f/+^Postn^+/+^* mice, we found that JNK phosphorylation was significantly decreased in the myocardium of *CreDSP^f/+^Postn^−/−^* mice, and this reduction was also observed in the group with AAV‐mediated cardiac fibroblast‐targeted deletion of *Postn* (Figure 4G and Figure S6A). Consistent with these results, rmPOSTN and rhPOSTN treatment increased JNK phosphorylation in NMCMs and AC16 cells transfected with si*DSP*, respectively (Figure 4H,I). To explore whether POSTN activated necroptosis through the integrin‐JNK axis, NMCMs transfected with si*DSP* were treated with an integrin αvβ3/αvβ5 neutralizing antibody or si*Itgβ3/β5* along with rmPOSTN (Figure S6B,C). The αvβ3/αvβ5 neutralizing antibody or si*Itgβ3/β5* markedly suppressed the phosphorylation of JNK, RIP3, and MLKL triggered by rmPOSTN (Figure 4J and Figure S6D). Furthermore, treatment with PD98059, a MAPKK inhibitor, or SP600125, a specific JNK inhibitor, effectively reversed necroptosis induced by rmPOSTN in NMCMs transfected with si*DSP* (Figure 4K). Moreover, treatment with Necrostatin‐1 (Nec‐1), a necroptosis inhibitor, further confirmed the cascading relationship between JNK and RIP3‐MLKL signaling pathways (Figure 4L). Similar results were obtained in AC16 cells (Figure 4M–O and Figure S6E–G). Collectively, these results demonstrate that POSTN binds to integrin receptors to activate the JNK signaling, thereby promoting the phosphorylation of RIP3 and MLKL as well as enhancing cardiomyocyte necroptosis in ACM mice.

### 2.5 POSTN Promotes CCL3 Expression Through the Integrin‐JNK‐ETS2 Signaling in Cardiomyocytes

**FIGURE 5:**
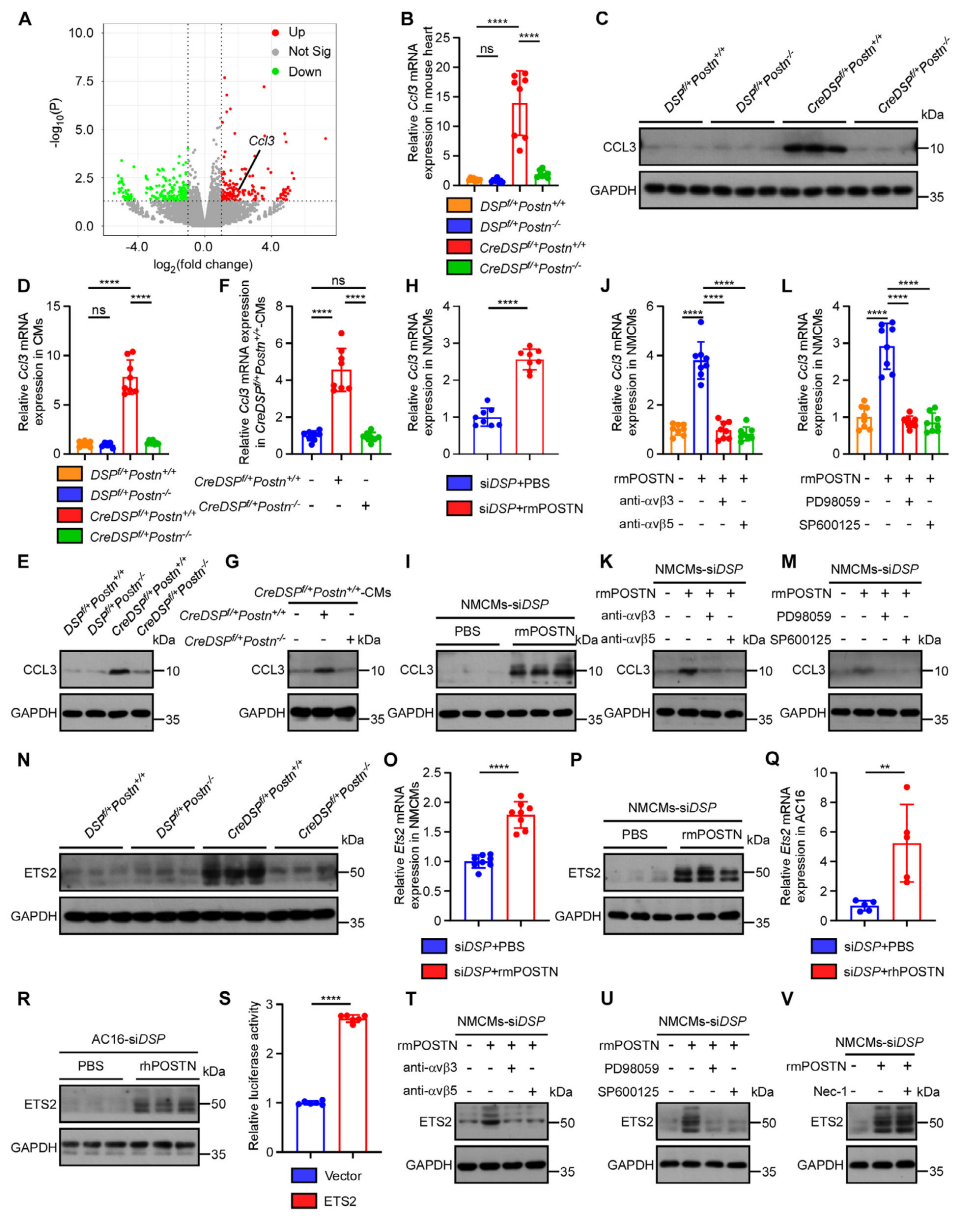
Integrin‐JNK‐ETS2 signaling mediates POSTN‐induced CCL3 upregulation in cardiomyocytes. (A) Differential gene expression from RNA‐seq analysis in the myocardium of *CreDSP^f/+^Postn^−/−^* mice compared to *CreDSP^f/+^Postn^+/+^* mice (*n* = 3). (B,C) Relative mRNA (B) (*n* = 8) and protein (C) levels of CCL3 in the myocardium of WT and *DSP*‐ACM mice with or without POSTN deficiency. (D,E) Relative mRNA (D) (*n* = 8) and protein (E) levels of CCL3 in CMs derived from WT and *DSP*‐ACM mice with or without POSTN deficiency. (F,G) Relative mRNA (F) (*n* = 8) and protein (G) levels of CCL3 in CMs isolated from *CreDSP^f/+^Postn^+/+^* mice, which were cultured alone or co‐cultured indirectly with CFs isolated from *CreDSP^f/+^Postn^+/+^* mice and *CreDSP^f/+^Postn^−/−^* mice. (H,I) Relative mRNA (H) (*n* = 8) and protein levels (I) of CCL3 in NMCMs transfected with si*DSP* in the presence or absence of rmPOSTN. (J–M) Relative mRNA (*n* = 8) and protein levels of CCL3 in NMCMs transfected with si*DSP* in the presence or absence of rmPOSTN, as well as αvβ3 or αvβ5 integrin neutralizing antibody (J,K), PD98059 or SP600125 (L,M). (N) The protein levels of ETS2 in the myocardium of WT and *DSP*‐ACM mice with or without POSTN deficiency. (O,P) Relative mRNA (O) (*n* = 8) and protein (P) levels of ETS2 in NMCMs transfected with si*DSP* in the presence or absence of rmPOSTN. (Q,R) Relative mRNA (Q) (*n* = 5) and protein (R) levels of ETS2 in AC16 cells transfected with si*DSP* in the presence or absence of rhPOSTN. (S) Dual luciferase activity assay of HEK293 cells co‐transfected with the *Ets2* and *Ccl3* (*n* = 6). (T–V) Western blot analysis of ETS2 in NMCMs transfected with si*DSP* in the presence or absence of rmPOSTN, as well as αvβ3 or αvβ5 integrin neutralizing antibody (T), PD98059 or SP600125 (U), and Nec‐1 (V). Statistical analyses were performed by one‐way ANOVA analysis followed by Tukey's multiple comparison tests (B,D,F,J,L), unpaired two‐tailed Student's *t*‐test (H,O,Q,S). Data are mean±SEM. ^**^ *p* < 0.01, ^****^ *p* < 0.0001. ns, no significance.

As a proinflammatory type of cell death, necroptosis leads to the upregulation of damage‐associated molecular patterns (DAMPs), proinflammatory cytokines, and chemokines [31, 32, 33]. RNA sequencing (RNA‐seq) of the myocardium from *CreDSP^f/+^Postn^+/+^* mice and *CreDSP^f/+^Postn^−/−^* mice revealed a significant decrease of *Ccl3*, a chemokine associated with necroptosis and inflammation, in *CreDSP^f/+^Postn^−/−^* mice (Figure 5A). Further analysis confirmed that POSTN deficiency markedly downregulated the mRNA and protein levels of CCL3 in the myocardium of ACM mice (Figure 5B,C and Figure S7A). To identify the cellular source of CCL3, we isolated CMs and NCMs from ACM mice. qRT‐PCR and Western blot analyses showed that *Ccl3* mRNA and CCL3 protein levels were significantly higher in CMs than NCMs (Figure S7B,C). Further quantification of isolated CMs, macrophages, and CFs revealed that both *Ccl3* mRNA and CCL3 protein levels were highest in cardiomyocytes, compared to macrophages and CFs (Figure S7D,E). Consistently, immunofluorescence and RNA‐FISH analyses of the ACM myocardium confirmed that CCL3 was predominantly localized to cTNT‐positive CMs (Figure S7F,G). Moreover, POSTN deficiency significantly reduced the mRNA and protein levels of CCL3 in the CMs of ACM mice (Figure 5D,E). In the in vitro co‐culture system, CMs derived from *CreDSP^f/+^Postn^+/+^* mice exhibited elevated CCL3 expression when co‐cultured with CFs isolated from *CreDSP^f/+^Postn^+/+^* mice compared to *CreDSP^f/+^Postn^−/−^* mice (Figure 5F,G). Treatment of rmPOSTN or rhPOSTN significantly increased CCL3 mRNA and protein levels in NMCMs and AC16 cells treated with si*DSP* (Figure 5H,I and Figure S7H,I). Furthermore, the αvβ3/αvβ5 neutralizing antibody or si*Itgβ3/β5* remarkably suppressed the expression of CCL3 induced by rmPOSTN in NMCMs treated with si*DSP* (Figure 5J,K and Figure S7J). Treatment with PD98059 or SP600125 significantly decreased CCL3 expression induced by rmPOSTN in NMCMs with si*DSP* (Figure 5L,M). Consistently, blocking integrin receptors or JNK signaling reduced CCL3 levels in AC16 cells treated with si*DSP* and rhPOSTN (Figure S7K–O), indicating that integrin‐JNK axis, which mediates cardiomyocyte necroptosis, is a key contributor to CCL3 upregulation.

To further investigate the transcription factors that regulate CCL3 expression, we analyzed alterations in downstream effectors within the myocardium of *CreDSP^f/+^Postn^+/+^* mice in response to JNK signaling pathways. The level of ETS2 was markedly decreased in the myocardium of *Postn*‐deficient ACM mice, while no significant changes were observed in other JNK‐regulated transcription factors (Figure 5N and Figure S8A,B). qPCR analysis further demonstrated that *Ets2* mRNA was significantly reduced in the isolated cardiomyocytes from *CreDSP^f/+^Postn^−/−^* mice compared to *CreDSP^f/+^Postn^+/+^* mice (Figure S8C). Consistently, rmPOSTN or rhPOSTN treatment increased ETS2 mRNA and protein levels in NMCMs and AC16 cells transfected with si*DSP* (Figure 5O–R and Figure S8D). Dual‐luciferase reporter analysis indicated that ETS2 transactivated the *Ccl3* promoter in AC16 cells transfected with si*DSP* (Figure 5S). We further confirmed that inhibition of integrin receptors or JNK signaling, but not cardiomyocyte necroptosis, reduced the POSTN‐induced increase of ETS2 levels in NMCMs and AC16 cells treated with si*DSP* (Figure 5T–V and Figure S8E–I). These data support a role for JNK signaling in POSTN‐induced ETS2 upregulation, which in turn promotes CCL3 expression in cardiomyocytes.

### 2.6 CCL3‐CCR5‐NF‐κB Signaling Induces Cardiac Fibroblast Activation and POSTN Expression

**FIGURE 6:**
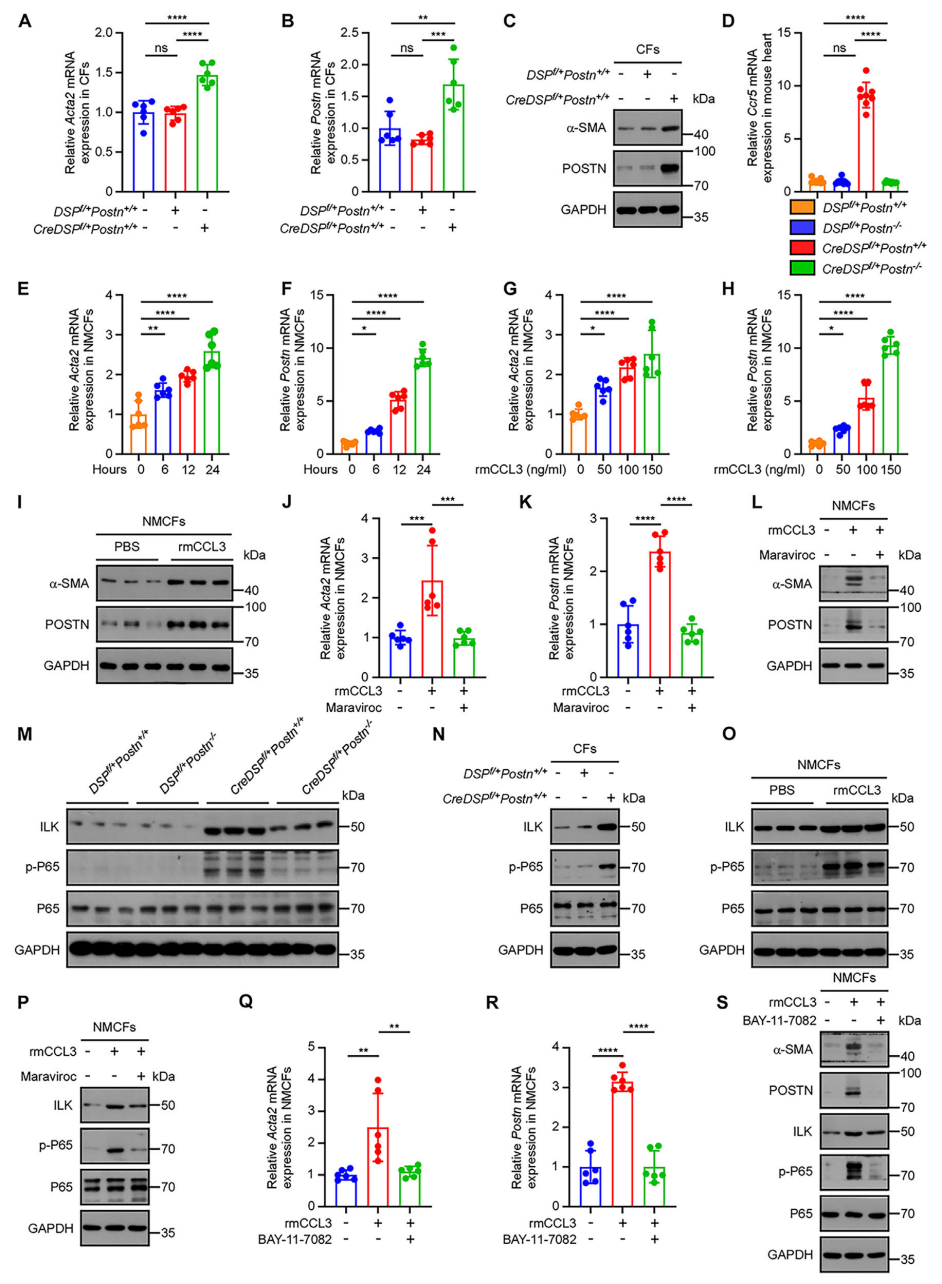
CCL3‐CCR5‐NF‐κB axis mediates cardiac fibroblast activation and POSTN expression. (A–C) Relative mRNA (A,B) (*n* = 6) and protein (C) levels of α‐SMA and POSTN in CFs derived from *DSP^f/+^Postn^+/+^* mice, which were cultured alone or co‐cultured indirectly with CMs derived from *DSP^f/+^Postn^+/+^* mice or *CreDSP^f/+^Postn^+/+^* mice. (D) Relative mRNA levels of *Ccr5* in the myocardium of WT and *DSP*‐ACM mice with or without POSTN deficiency (*n* = 8). (E–H) Relative mRNA expression of *Acta2* (E,G) and *Postn* (F,H) in NMCFs treated with rmCCL3 for the indicated time or indicated doses for 24 h (*n* = 6). (I) The protein levels of α‐SMA and POSTN in NMCFs treated with PBS or rmCCL3. (J–L) Relative mRNA (J,K) (*n* = 6) and protein (L) levels of α‐SMA and POSTN in NMCFs treated with or without rmCCL3 in the presence or absence of Maraviroc. (M) The protein levels of ILK, p‐P65, and P65 in the myocardium of WT and *DSP*‐ACM mice with or without POSTN deficiency. (N) Western blot analysis of ILK, p‐P65, and P65 in CFs derived from *DSP^f/+^Postn^+/+^* mice, which were cultured alone or co‐cultured indirectly with CMs derived from *DSP^f/+^Postn^+/+^* mice or *CreDSP^f/+^Postn^+/+^* mice. (O,P) The protein levels of ILK, p‐P65, and P65 in NMCFs treated with rmCCL3 (O) and in the presence or absence of Maraviroc (P). (Q,R) Relative mRNA (*n* = 6) levels of *Acta2* (Q) and *Postn* (R) in NMCFs treated with or without rmCCL3 in the presence or absence of BAY‐11‐7082. (S) Western blot analysis of α‐SMA, POSTN, ILK, p‐P65 and P65 in NMCFs treated with or without rmCCL3 in the presence or absence of BAY‐11‐7082. Statistical analyses were performed by one‐way ANOVA analysis followed by Tukey's multiple comparison tests (A,B,D,E–H,J,K,Q,R). Data are mean±SEM. ^*^ *p* < 0.05, ^**^ *p* < 0.01, ^***^ *p* < 0.001, ^****^ *p* < 0.0001. ns, no significance.

Our in vitro co‐culture system revealed that CFs derived from *DSP^f/+^ Postn^+/+^* mice exhibited elevated α‐SMA and POSTN expression when co‐cultured with CMs isolated from *CreDSP^f/+^Postn^+/+^* mice compared to those from *DSP^f/+^Postn^+/+^* mice (Figure 6A–C). qRT‐PCR analysis showed that the level of *Ccr5*, a receptor for CCL3, was significantly decreased in the myocardium of *CreDSP^f/+^Postn^−/−^* mice compared with *CreDSP^f/+^Postn^+/+^* mice (Figure 6D), while there was no significant difference in the expression of *Ccr1* and *Ccr3* (Figure S9A). To investigate the role of CCL3, we treated isolated neonatal mouse cardiac fibroblasts (NMCFs) with rmCCL3. rmCCL3 stimulation significantly promoted fibroblast activation, leading to a time‐ and dose‐dependent increase in the mRNA levels of *Acta2* and *Postn* (Figure 6E–H). Western blot analysis confirmed that rmCCL3 significantly elevated α‐SMA and POSTN levels in NMCFs, indicating that CCL3 in turn induces fibroblast activation and POSTN expression (Figure 6I). Furthermore, treatment with Maraviroc, a potent CCR5 inhibitor, effectively reversed fibroblast activation and POSTN expression induced by rmCCL3 or co‐cultured with *CreDSP^f/+^Postn^+/+^* CMs (Figure 6J–L and Figure S9B–D).

Accumulating evidence implies that NF‐κB signaling is induced by CCL3 and plays a crucial role in cardiac fibroblast activation [34, 35]. In vivo, the levels of ILK and p‐P65 were significantly decreased in the myocardium of *CreDSP^f/+^Postn^−/−^* mice compared to *CreDSP^f/+^Postn^+/+^* mice (Figure 6M). In our in vitro co‐culture system, CFs derived from *DSP^f/+^Postn^+/+^* mice exhibited elevated ILK and p‐P65 levels when co‐cultured with CMs isolated from ACM mice (*CreDSP^f/+^Postn^+/+^*) compared to the control mice (*DSP^f/+^Postn^+/+^*) (Figure 6N). Consistently, rmCCL3 treatment strongly increased ILK and p‐P65 levels in the NMCFs (Figure 6O). Maraviroc treatment significantly blocked the rmCCL3‐induced increase in ILK and p‐P65 levels (Figure 6P). Moreover, treatment with BAY‐11‐7082, an NF‐κB‐signaling inhibitor, in NMCFs decreased α‐SMA and POSTN levels and inhibited NF‐κB signaling activation induced by CCL3 stimulation (Figure 6Q–S). Similar results were observed in CFs co‐cultured with CMs isolated from *CreDSP^f/+^Postn^+/+^* mice (Figure S9E–G). These findings suggest that the CCL3‐CCR5‐NF‐κB signaling plays a pivotal role in cardiac fibroblast activation and POSTN expression. Importantly, this cascade establishes that CCL3‐induced NF‐κB signaling activation drives POSTN expression in CFs, which further facilitates cardiomyocyte necroptosis and CCL3 upregulation, contributing to ACM progression.

### 2.7 POSTN is Positively Correlated With Cardiomyocyte Necroptosis and CCL3 Upregulation in the Myocardium of Patients and Mice With ACM

**FIGURE 7:**
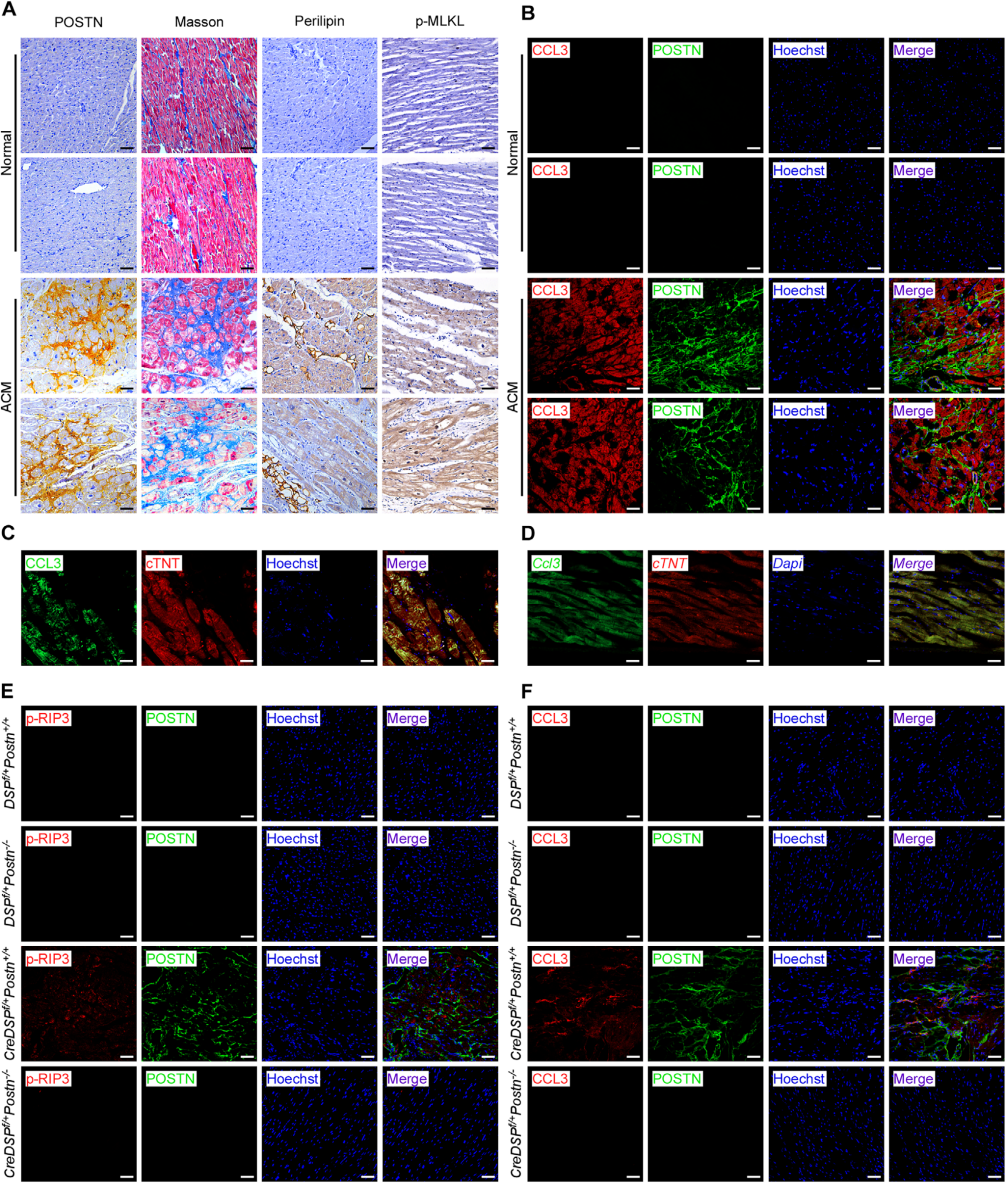
POSTN expression positively correlates with cardiomyocyte necroptosis in clinical and murine ACM hearts. (A) Masson's Trichrome staining, immunohistochemical staining of POSTN, Perilipin, and p‐MLKL in cardiac sections from normal controls and patients with ACM. Scale bars, 50 µm. (B) Immunofluorescence co‐staining of CCL3 (red) and POSTN (green) in cardiac sections from normal controls and patients with ACM. Scale bars, 50 µm. (C) Immunofluorescence co‐staining of CCL3 (green) and cTNT (red) in cardiac sections from patients with ACM. Scale bars, 50 µm. (D) RNA‐FISH staining of *Ccl3* and cardiomyocytes (*cTNT*) in cardiac sections of patients with ACM. Scale bars, 50 µm. (E,F) Immunofluorescence co‐staining of p‐RIP3 (E) or CCL3 (F) (red) and POSTN (green) in cardiac sections from WT and *DSP*‐ACM mice with or without POSTN deficiency. Scale bars, 50 µm.

To further elucidate the role of POSTN in ACM progression, we detected POSTN levels in the hearts of healthy controls and patients with ACM. Compared to the control group, immunohistochemical staining revealed a significant increase of POSTN in the myocardium of patients with ACM (Figure 7A). Additionally, upregulation of POSTN was related to the severity of fibrofatty replacement and cardiomyocyte necroptosis in patients with ACM (Figure 7A). Immunofluorescence staining further verified increased levels of POSTN and CCL3 in hearts of patients with ACM (Figure 7B). Importantly, Immunofluorescence staining and RNA‐FISH of ACM patient hearts confirmed that CCL3 was mainly derived from c‐TNT^+^ cardiomyocytes (Figure 7C,D). Similarly, POSTN, p‐RIP3, and CCL3 were spatially correlated in the myocardium of *CreDSP^f/+^Postn^+/+^* mice compared with *CreDSP^f/+^Postn^−/−^* mice (Figure 7E,F). These findings suggest a positive correlation between POSTN expression and cardiomyocyte necroptosis as well as cardiac fibrosis in ACM.

### 2.8 Targeting the JNK‐CCL3‐CCR5 Axis Attenuates Cardiac Remodeling and Necroptosis in ACM Mice

To define the temporal role of POSTN‐mediated signaling during ACM progression in mice, we first examined mice at 4 months of age. At this stage, no significant differences were observed among the experimental groups in cardiac morphology, heart failure markers, fibrosis, necroptosis or activation of the JNK‐ETS2‐CCL3 signaling axis. In contrast, at 8 months of age, *CreDSP^f/+^Postn^+/+^* mice developed marked cardiac fibrosis, cardiomyocyte necroptosis, and robust activation of the JNK‐ETS2‐CCL3 pathway (Figure S10A–I). These findings indicate that the POSTN‐CCL3 axis may be not critical to initiate ACM, which is primarily driven by desmosomal dysfunction. Instead, it is activated during disease progression and might act as a pathogenic amplification loop that contributes to cardiac fibrosis and cardiomyocyte necroptosis.

**FIGURE 8:**
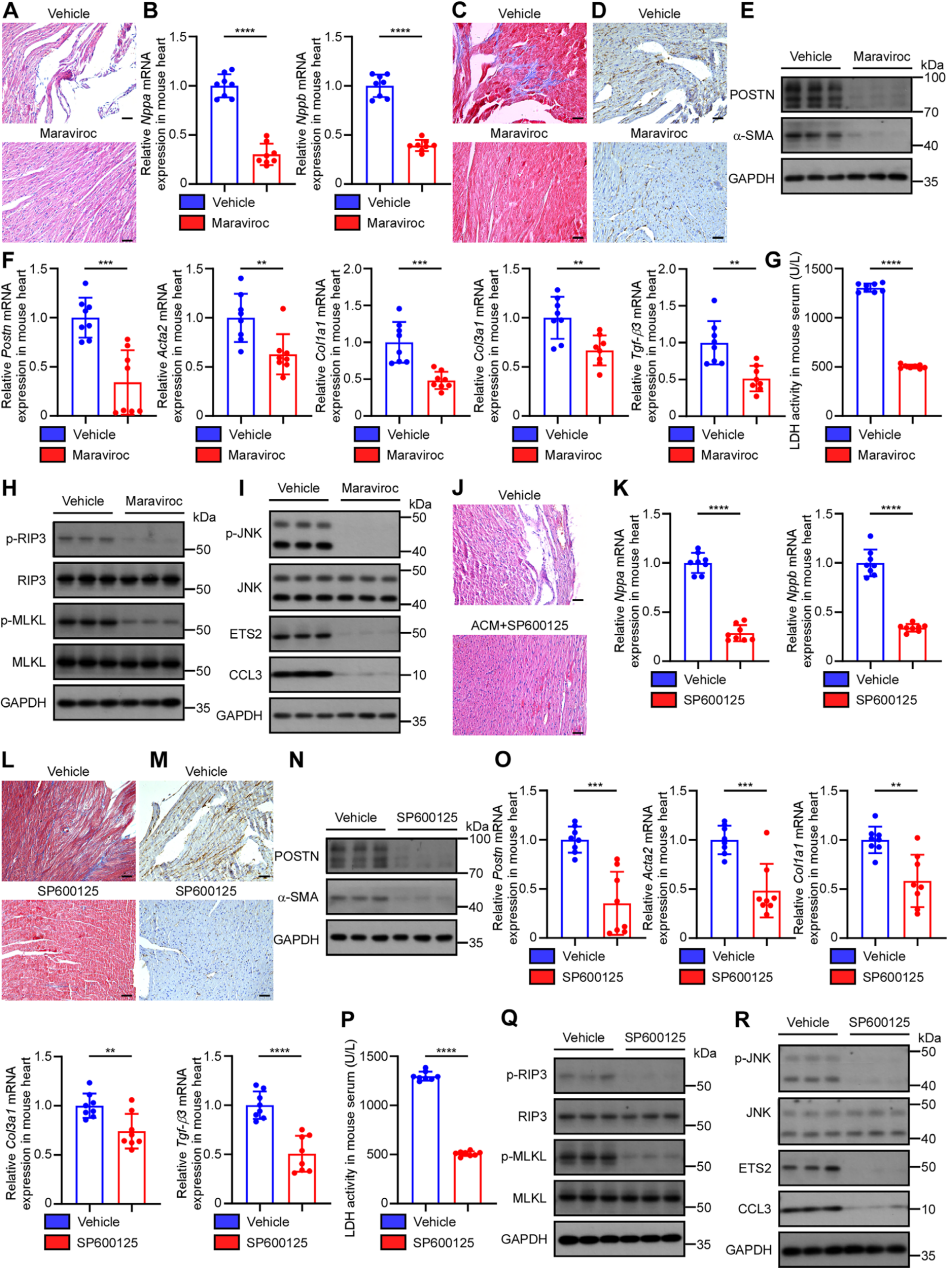
Pharmacological inhibition of the JNK‐CCL3‐CCR5 axis attenuates cardiac remodeling and necroptosis in ACM mice. (A) H&E staining in cardiac sections from Vehicle‐ and Maraviroc‐treated ACM mice. Scale bars, 50 µm. (B) mRNA levels of *Nppa* and *Nppb* in the myocardium of Vehicle‐ and Maraviroc‐treated ACM mice (*n* = 8). (C,D) Masson's Trichrome staining (C) and immunohistochemical staining of α‐SMA (D) in cardiac sections from Vehicle‐ and Maraviroc‐treated ACM mice. Scale bars, 50 µm. (E) Western blot analysis of POSTN and α‐SMA in the cardiac tissue lysates of Vehicle‐ and Maraviroc‐treated ACM mice. (F) mRNA levels of *Postn*, *Acta2*, *Col1a1*, *Col3a1*, and *Tgfβ3* in the myocardium of Vehicle‐ and Maraviroc‐treated ACM mice (*n* = 8). (G) The activities of LDH in the serum isolated from Vehicle‐ and Maraviroc‐treated ACM mice (*n* = 8). (H) Western blot analysis of the indicated proteins associated with necroptosis in the myocardium derived from Vehicle‐ and Maraviroc‐treated ACM mice. (I) The protein levels of p‐JNK, JNK, ETS2, and CCL3 in the myocardium derived from Vehicle‐ and Maraviroc‐treated ACM mice. (J) H&E staining in cardiac sections from Vehicle‐ and SP600125‐treated ACM mice. Scale bars, 50 µm. (K) mRNA levels of *Nppa* and *Nppb* in the myocardium of Vehicle‐ and SP600125‐treated ACM mice (*n* = 8). (L,M) Masson's Trichrome staining (L) and immunohistochemical staining of α‐SMA (M) in cardiac sections from Vehicle‐ and SP600125‐treated ACM mice. Scale bars, 50 µm. (N) Western blot analysis of POSTN and α‐SMA in the cardiac tissue lysates of Vehicle‐ and SP600125‐treated ACM mice. (O) mRNA levels of *Postn*, *Acta2*, *Col1a1*, *Col3a1* and *Tgfβ3* in the myocardium of Vehicle‐ and SP600125‐treated ACM mice (*n* = 8). (P) The activities of LDH in the serum isolated from Vehicle‐ and SP600125‐treated ACM mice (*n* = 8). (Q) Western blot analysis of the indicated proteins associated with necroptosis in the myocardium derived from Vehicle‐ and SP600125‐treated ACM mice. (R) The protein levels of p‐JNK, JNK, ETS2, and CCL3 in the myocardium derived from Vehicle‐ and SP600125‐treated ACM mice. Statistical analyses were performed by unpaired two‐tailed Student's *t*‐test (B,F,G,K,O,P). Data are mean±SEM. ^**^ *p* < 0.01, ^***^ *p* < 0.001, ^****^ *p* < 0.0001.

Given the pathogenic role of this feedforward circuit and the lack of a specific inhibitor of POSTN, we next selected inhibitors of CCR5 and JNK to investigate whether pharmacological blockade of the POSTN‐JNK‐CCL3‐CCR5 axis could attenuate the progression of ACM in mice. 5‐month‐old *CreDSP^f/+^Postn^+/+^* mice were treated with the FDA‐approved CCR5 antagonist Maraviroc. Maraviroc markedly improved cardiac histopathology (Figure 8A) and significantly reduced the expression of the heart failure markers *Nppa* and *Nppb* (Figure 8B). Cardiac fibrosis was also significantly attenuated, as evidenced by reduced collagen deposition, POSTN and α‐SMA expression, and the mRNA expression of *Postn* and profibrotic genes (Figure 8C–F). Maraviroc attenuated cardiomyocyte injury and necroptosis, as indicated by reduced serum LDH levels and decreased phosphorylation of RIP3 and MLKL (Figure 8G,H). Moreover, Maraviroc suppressed activation of the JNK‐ETS2‐CCL3 signaling axis, consistent with attenuation of the proposed pathogenic feedforward loop (Figure 8I). We further treated the 5‐month‐old *CreDSP^f/+^Postn^+/+^* mice with the JNK inhibitor SP600125 and found that SP600125 treatment produced comparable protective effects in ACM mice, including attenuation of cardiac remodeling and fibrosis, suppression of cardiomyocyte necroptosis, and inhibition of POSTN‐JNK‐ETS2‐CCL3 signaling (Figure 8J–R).

Collectively, these results support the potential of pharmacological inhibition of JNK or CCR5 to attenuate ACM‐associated cardiac remodeling, fibrosis, and cardiomyocyte necroptosis, accompanied by suppression of the POSTN‐JNK‐ETS2‐CCL3 signaling cascade. Notably, these protective effects are broadly consistent with those observed in POSTN‐deficient mice, providing in vivo evidence supporting a pathogenic role for the POSTN‐JNK‐ETS2‐CCL3 signaling in ACM progression.

## 3 Discussion

In this study, we offer new perspectives on the role of POSTN in the progression of ACM. Our findings highlight POSTN as an important regulator of cardiomyocyte necroptosis and cardiac fibrosis. Importantly, we demonstrate that POSTN promotes cardiomyocyte necroptosis via the integrin‐JNK‐RIP3‐MLKL axis and increases CCL3 expression, which is associated with cardiac fibroblast activation and POSTN expression through the CCL3‐CCR5‐NF‐κB signaling. Moreover, the interactions mediated by POSTN and CCL3 between cardiomyocytes and fibroblasts not only promote cardiomyocyte necroptosis but also contribute to fibrotic remodeling and ACM progression (Figure S11).

In ACM, cardiomyocyte death is a fundamental pathological feature that contributes to disease progression [36, 37]. Cardiomyocyte death has been identified as a key pathological event in myocardial injury in ACM patients, leading to increased DNA damage and progressive disruption of nuclear envelope integrity, followed by myocardial loss and fibrofatty replacement, contributing to heart failure and sudden death in patients with ACM [38, 39, 40, 41, 42]. Cardiomyocyte death occurs through multiple mechanisms, including necrosis [43, 44], apoptosis [45, 46], and autophagy [47]. While prior studies have linked apoptosis and other programmed cell death pathways to ACM, our study highlights a previously underestimated role of necroptosis in ACM progression. We found that POSTN promotes cardiomyocyte necroptosis through the integrin‐JNK‐RIP3‐MLKL axis. The upregulation of POSTN in the myocardium of both *DSP*‐mutant ACM mice and patients with ACM, coupled with a significant reduction in LDH activity and levels of p‐RIP3, and p‐MLKL in POSTN‐deficient ACM mice supports a role for POSTN in promoting cardiomyocyte necroptosis. Treatment of neutralizing antibodies against integrins and inhibitors of JNK significantly attenuate POSTN‐induced cardiomyocyte necroptosis, suggesting that POSTN promotes cardiomyocyte necroptosis through the integrin‐JNK‐RIP3‐MLKL axis. These findings deepen our understanding of the molecular mechanisms contributing to ACM progression, identifying necroptosis as an important contributor to cardiomyocyte loss.

As cardiomyocytes die, the myocardium is replaced by fibrofatty tissue [41]. Fibrosis acts as a maladaptive repair mechanism where fibroblasts produce excessive ECM proteins, leading to the formation of fibrotic scars. This disrupts the normal myocardial structure and weakens cardiac function [48, 49]. POSTN plays an important role in the progression of fibrosis in diverse cardiovascular diseases, including myocardial infarction [50], heart failure [17], and hypertrophic cardiomyopathy [51]. POSTN facilitates fibrosis by promoting ECM stabilization, myofibroblast activation, and TGF‐β signaling [20, 52]. Its overexpression drives excessive scarring, contributing to organ dysfunction in fibrotic diseases [53]. Our findings reveal that POSTN deficiency reduces cardiac fibrosis in ACM mice. CCL3 induces cardiac fibroblast activation and POSTN expression through the CCL3‐CCR5‐NF‐κB signaling, thereby potentially amplifying fibrosis in ACM. These results advance our understanding of the mechanisms behind fibrofatty remodeling in ACM.

In ACM, cardiomyocyte and fibroblast interactions play a pivotal role in pathological remodeling [54]. Cardiomyocyte death releases pro‐inflammatory signals that activate fibroblasts [55, 56]. Activated fibroblasts proliferate and secrete ECM components, contributing to fibrosis. This pathological fibrotic structure disrupts myocardial architecture and electrical conduction, promoting arrhythmias and ventricular dysfunction [57]. Our results demonstrate that fibroblast‐derived POSTN promotes cardiomyocyte necroptosis through the integrin‐JNK‐RIP3‐MLKL axis and promotes CCL3 expression by the integrin‐JNK‐ETS2 signaling. In turn, increased CCL3 availability is associated with activation of fibroblasts and increased POSTN expression via the CCL3‐CCR5‐NF‐κB signaling. POSTN and CCL3 contribute to the crosstalk between cardiomyocytes and fibroblasts, creating a vicious cycle of cardiomyocyte necroptosis and cardiac fibrosis, thereby potentially amplifying ACM progression (Figure S11). Here, we further found that pharmacological inhibition of either JNK or CCR5 attenuates ACM‐associated cardiac remodeling, fibrosis and cardiomyocyte necroptosis. These findings suggest that disrupting the crosstalk between cardiomyocytes and fibroblasts may represent a potential therapeutic strategy for attenuating pathological remodeling in ACM.

Need to note, in this study we used a conditionally *DSP*‐knocked down mouse model, a well‐established model that recapitulates key features of ACM, including cardiomyocyte injury, fibrotic remodeling, and ventricular dysfunction. Within this context, we observed a marked upregulation of POSTN accompanied by activation of necroptotic signaling during disease progression. However, whether these changes are specific to DSP deficiency or represent a broader feature of desmosomal cardiomyopathy remains an open question and warrants further investigation. In addition, we found that cardiomyocytes are a predominant source of CCL3 in ACM myocardium; however, cardiomyocyte‐specific *Ccl3* deletion will be required to establish the functional necessity of cardiomyocyte‐derived CCL3 in ACM progression.

In summary, this study demonstrates the important role of cardiomyocyte necroptosis and fibroblast activation in the pathological remodeling of ACM. The reciprocal interactions between these two cell types, mediated by POSTN and CCL3, contribute to myocardial injury and fibrosis, establishing a feedforward signaling circuit that may amplify disease progression. Pharmacological targeting of JNK or CCR5 attenuated cardiac remodeling and necroptosis in ACM mice, supporting the therapeutic potential of interfering with the POSTN‐CCL3‐associated signaling network.

## 4 Experimental Section

### 4.1 Animals Studies

The ACM animal model, a heterozygous genetically modified mouse model featuring a cardiac‐restricted knockdown of the *DSP* gene, is achieved by crossing floxed *DSP* mice—carrying loxP sites flanking exon 2 of the *DSP* gene—with α‐MHC‐Cre mice that express Cre recombinase specifically in CMs [25]. Given the high embryonic lethality in *CreDSP^−/−^* mice, *CreDSP^f/+^* mice were utilized for subsequent investigations. The experimental mice (*DSP^f/+^Postn^+/+^* mice, *DSP^f/+^Postn^−/−^* mice, *CreDSP^f/+^Postn^+/+^* mice and *CreDSP^f/+^Postn^−/−^* mice) were acquired through the crossing of *CreDSP^f/+^* mice with *Postn^+/−^* mice. Cardiac‐restricted *DSP*‐deficient (*CreDSP^f/+^*) mice were kindly provided by Ali J. Marian at Baylor College of Medicine and maintained in the State Key Laboratory of Cardiovascular Disease, Fuwai Hospital. *Postn^+/−^* mice were obtained from Jackson Laboratory. Mice were maintained under specific pathogen‐free conditions at the Laboratory Animal Center of Xiamen University. Mice (7‐8‐month‐old) were analyzed for cardiac failure and fibrosis. The survival time of the mice was recorded for the survival curve analysis. The Animal Care and Use Committee of Xiamen University approved the animal experiments (Permit Number: XMULAC20200203).

### 4.2 In Vivo Pharmacological Interventions

For in vivo pharmacological interventions, 5‐month‐old ACM mice were administered the FDA‐approved CCR5 antagonist Maraviroc (MedChemExpress, HY‐13004) via daily oral administration at a dose of 50 mg/kg for 3 months. For JNK pathway inhibition, 5‐month‐old ACM mice received the JNK inhibitor SP600125 (MedChemExpress, HY‐12041) via intraperitoneal (i.p.) injection at a dose of 25 mg/kg. To minimize vehicle‐associated cumulative toxicity and procedural stress over the 3‐month regimen, SP600125 was administered intermittently three times weekly. Control mice were treated with the corresponding vehicles using identical schedules and administration routes.

### 4.3 AAV‐Mediated Cardiac Fibroblast‐Targeted Deletion of Postn

To specifically target *Postn* in cardiac fibroblasts, *CreDSP^f/+^Postn^f/f^* mice (C57BL/6J, 4 weeks old) received a single tail vein injection of AAV9 vectors expressing Cre recombinase under the control of the cardiac fibroblasts‐enriched *Tcf21* promoter (AAV9‐*Tcf21*‐Cre, 1.5 × 10^11^ viral genomes per mouse). The experimental endpoint was 8 months of age. Appropriate control mice received the corresponding AAV9‐negative control.

### 4.4 Cell Lines and Culture

The human cardiomyocyte cell line AC16 was purchased from the American Type Culture Collection (ATCC). CMs and non‐cardiomyocytes were isolated from adult mice myocardium following the procedure described by Matthew et al. [26]. Briefly, 8‐month‐old mice were anesthetized using Avertin (0.2 g/kg i.p., Sigma–Aldrich, T48402) and then disinfected with 75% ethanol. After severing the inferior vena cava, EDTA buffer was immediately injected into the right ventricle to remove the blood remaining in the heart. The descending aorta was securely clamped, followed by the left ventricle perfused with EDTA buffer and the perfusion buffer. Pre‐warmed collagenase buffer was subsequently introduced into the heart to facilitate tissue digestion. CMs and NCMs were isolated through sequential gravitational sedimentation techniques. For the isolation of NMCFs and neonatal mouse NMCMs, the hearts were rinsed with PBS containing 10% penicillin and streptomycin immediately after removal. The heart was treated with 0.25% trypsin. The isolated cells were subsequently gathered and prepared for further processing. Cells were cultured in DMEM medium (Yuanpei, L110KJ) supplemented with 1% penicillin‐streptomycin solution (Invitrogen, 15140122) and 10% fetal bovine serum (FBS) (Gibco) in a humid condition with 5% CO_2_ at 37°C.

### 4.5 Synthesis and Transfection of si*DSP*, si*Itgβ3* and si*Itgβ5*

The siRNAs targeting *DSP*, *Itgβ3, and Itgβ5* were designed and synthesized by GenePharma. NMCMs and AC16 cells were placed into 6‐well plates with serum‐starved DMEM medium. 100 µm of si‐negative control (siNC) or si*DSP*s (or *siItgβ3* or *siItgβ5)* was added when the cell reaching 60%–80% confluence with Lipofectamine 2000 reagent (Invitrogen, 11668027). Following 36 h of transduction, NMCMs, AC16 cells, and cardiac fibroblasts were treated with rmPOSTN or rhPOSTN protein (200 ng/ml) for 12 h, respectively. The sequences of siRNAs and the negative control were listed in Tables S1 and S2.

### 4.6 Human Specimen Collection

Human tissue collection and analysis were conducted following the guidelines of the Ethics Committee of Fuwai Hospital, Chinese Academy of Medical Sciences and Peking Union Medical College (Approval Number: 2013–496). Human tissue information was listed in Table S3.

### 4.7 Echocardiographic Measurements

Following inhalation of 2% isoflurane (RWD, R510‐22), mice underwent transthoracic echocardiography using the High‐Resolution Imaging System (Visual Sonics, Vevo2100) with mice's body temperature within 36.9°C to 37.3°C and heart rates between 400 and 550 bpm. The left ventricular ejection fraction (EF) and fractional shortening (FS) were analyzed with software provided by the manufacturer based on M‐mode recordings.

### 4.8 Electrocardiogram Detection

Mice were subjected to electrocardiogram detection using the Biological Signal Acquisition and Analysis System (AD Instruments, ML4856) to evaluate cardiac function. The electrodes of the electrocardiogram detectors were attached to the limbs of the anesthetized mice. Dynamic electrocardiogram images and measurements were recorded after the images stabilized.

### 4.9 Mouse Treadmill Running

The mice (8‐month‐old) were subjected to 10‐min treadmill adaptive training at a speed of 6 m/s. The treadmill's electric shock intensity was set to 0.4 mA. The mice engaged in the treadmill exercise with a uniform variable speed, starting at 6 m/min, increasing by 2 m/min every 3 min, and maintaining a steady acceleration to 18 m/min. Mice were shocked five times as the standard for exhaustion. Cardiac function of mice was evaluated according to the recorded training time.

### 4.10 Quantification and Statistical Analysis

All data were shown as the mean ± SEM. Prism software (GraphPad 8.0) was used to perform statistical analyses. The sample size per group was shown in the figure legends. In the figures, statistical significance is indicated as follows: ^*^ *p* < 0.05, ^**^ *p* < 0.01, ^***^ *p* < 0.001, ^****^ *p* < 0.0001. Meanwhile, ns represents no significant difference. Nonpaired two‐tailed Student's *t*‐test was employed to make a comparison of the data between two groups. For the analysis of multiple groups, one‐way ANOVA analysis, which was based on Tukey's post‐hoc multi‐comparison test, was utilized.

## Author Contributions

T.W., R.L., D.C., J.S., and G.O. designed the study. T.W. and R.L. arranged experiments and analyzed the results. T.W., R.L., X.Z., D.C., J.Y., M.L., and Y.W. conducted animal experiments and analyzed the results. T.W., R.L., J.Y., Y.Y., Y.T., and H.Y. performed cellular experiments. X.Z., M.L., and J.S. collected human specimens. T.W., R.L., and G.O. wrote and finalized the article. All authors have made contributions to this work.

## Conflicts of Interest

The authors declare no conflicts of interest.

## Supporting information




**Supporting File**: advs77670‐sup‐0001‐SuppMat.docx.

## Data Availability

All data produced or examined in this research are presented in Figures 1, 2, 3, 4, 5, 6, 7, 8 and Figures S1–S11. Supporting datasets that reinforce the study's conclusions can be obtained from the corresponding author upon reasonable request.
