# Supplementary material for: Periostin‐CCL3 Feedforward Signaling Loop Promotes Cardiac Fibrosis and Cardiomyocyte Necroptosis in Arrhythmogenic Cardiomyopathy
Source: Adv Sci (Weinh). 2026 Sep 10:e77670. Online ahead of print. doi: 10.1002/advs.77670 (PMC13562787; doi:10.1002/advs.77670)
Supplement: Supplementary file 1 [file ADVS-9999-e77670-s001.docx]

**Supplemental material**

**Supplementary methods**

Hematoxylin and eosin staining, Masson’s Trichrome staining and Sirius Red staining

Immunohistochemical staining

Immunofluorescence staining

Lactate dehydrogenase activity detection assay (LDH assay)

Western blot

Quantitative real-time polymerase chain reaction (qRT-PCR)

Fluorescence in situ hybridization (FISH)

Luciferase reporter assay

Enzyme-linked immunosorbent assay (ELISA)

Propidium iodide staining (PI staining)

**Supplementary figures**

**Figure S1.** POSTN is significantly elevated in the myocardium and serum and facilitates fibroblast activation

**Figure S2.** POSTN deficiency rescues myocardium loss and dysfunction but does not restore desmosomal structural defects in ACM mice

**Figure S3.** POSTN deficiency reduces inflammation in the myocardium of *DSP*-ACM mice

**Figure S4.** POSTN deficiency ameliorates cardiac fibrosis in *DSP*-ACM mice

**Figure S5.** POSTN deficiency attenuates cardiomyocyte necroptosis

**Figure S6.** POSTN deficiency attenuates cardiomyocyte necroptosis via the integrin-JNK-RIP3-MLKL axis.

**Figure S7.** Cardiomyocytes are the predominant cellular source of CCL3

**Figure S8.** POSTN upregulates CCL3 level by the integrin-JNK-ETS2 signaling

**Figure S9.** CCL3 promotes cardiac fibroblast activation and POSTN expression by activating the CCR5-NF-κB signaling

**Figure S10.** POSTN deficiency doesn’t significantly influence cardiac fibrosis and cardiomyocyte necroptosis in 4-month-old ACM mice

**Figure S11.** The graphic illustration on the role of POSTN-CCL3 loop in *DSP*-ACM progression

**Supplementary tables**

**Table S1.** The sequences of siRNAs for *DSP, Itgβ3 and Itgβ5*

**Table S2.** The sequences of negative control for siRNAs

**Table S3.** Individual clinical and genetic characteristics of patients

**Table S4.** Key resources table

**Table S5.** Sequences of mouse oligonucleotide primers used for qRT- PCR

**Table S6.** Primer sequences for FISH

**Table S7.** Primers of plasmids for luciferase reporting assay

**Supplementary methods**

*Hematoxylin and eosin staining, Masson’s Trichrome staining and Sirius Red staining*: Histological analyses of cardiac sections were conducted by using the Hematoxylin and eosin staining kit (Beyotime, C0105S), Masson’s Trichrome staining assay kit (Servicebio, G1006) and Sirius Red staining kit (Saint-bio, R21890) following the manufacturer’s instructions and quantified by Image J analysis software.

*Immunohistochemical staining*: Deparaffinized cardiac sections were carefully positioned in antigen retrieval solution (0.01 M sodium citrate buffer) at 98°C for 10 minutes. Subsequently, they were cooled at room temperature and blocked with goat serum. The treated slides were incubated with the primary antibodies at 4°C overnight, followed by incubating with corresponding secondary antibodies at room temperature for two hours. Then slides were stained with DAB (MXB, DAB-2031) after washing for 30 minutes. Filtered hematoxylin (Auragene Bioscience, P017IH/P032IH) was applied to stain nuclei. The cardiac sections were examined under the upright microscope (Leica, DM4B).

*Immunofluorescence staining*: For cardiac sections, dehydrated slides were incubated with blocking solution (3% BSA and 0.4% Triton X-100 in PBS), followed by incubating with the primary antibodies at 4°C overnight under humidified conditions, and then treated with fluorescent conjugated secondary antibodies (1:400, Abcam, ab150075/150117& 1:400, Thermo Scientific, A48263) for 1 hour. The cardiac tissue sections were counterstained with Hoechst. Slides were examined under a confocal microscope (Zeiss, LSM 780).

For cells, cells on the coverslip were fixed in 4% paraformaldehyde for half an hour and then rinsed 3 times with PBS. After blocking with 10% FBS for 2 hours, slides were incubated overnight at 4°C with primary antibodies, and then treated with secondary antibodies for 2 hours. Cell nuclei were stained with Hoechst. Slides were examined under a confocal microscope (Zeiss, LSM 780).

*Lactate dehydrogenase activity detection assay (LDH assay)*: The serum or culture supernatant extracted from mice and cells was detected by the LDH detection kit (Nanjing Jiancheng, A020-2-2). Double distilled water, 0.2 μmol/ml pyruvate standard solution, matrix buffer, coenzyme I, 2, 4-dinitrophenylhydrazine, and 0.4 mol/L NaOH solution were added to the samples in accordance with the manufacturer’s instructions. The samples were tested with absorbance at 450 nm using a microplate reader (Bio Tek).

*Western blot*: Total proteins were obtained from myocardium or cells using RIPA lysis buffer containing phosphatase and protease inhibitors. Protein concentration was measured with Pierce™ BCA Protein Assay Kit (Thermo Scientific, 23227), and equal amounts were loaded onto 10-12% SDS-PAGE gels, followed by transfer to PVDF membranes (Millipore, IPVH00010). Membranes were blocked with 5% BSA and incubated overnight at 4°C with primary antibodies (listed in Table S4), then treated with HRP-conjugated secondary antibodies for 2 hours at room temperature (1:5,000, Thermo Scientific, 31430/31460/31470). Protein bands were visualized using X-ray films.

*Quantitative real-time polymerase chain reaction (qRT-PCR)*: Using TRIzol reagent (Invitrogen, 15596018CN), total RNAs were extracted from tissues and cells following the manufacturer’s guidelines. Complemental DNA was synthesized by 5×All-In-One RT MasterMix (Applied Biological Materials, G490). qRT-PCR was assessed using the fluorescent dye SYBR Green (TransGen Biotech, AQ131-04). Gapdh and S18 served as internal controls. Primer sequences were listed in Table S5.

*Fluorescence in situ hybridization (FISH)*: Fluorescence *in Situ* Hybridization (FISH) was carried out in accordance with the Fluorescence *in Situ* Hybridization Kit (GenePharma, F26501/100). Deparaffinized slides were incubated with protease K working fluid at 37°C for 20 minutes. Subsequently, they were washed 3 times with 2×Buffer C. After dehydrating with increasing concentrations of alcohol, slides were incubated with the prepared denaturing solution for 8 minutes at 78°C, followed by further dehydrating with alcohol. Slides were incubated with a hybridization solution containing the specific probe for *Vimentin*, *Postn*, *Ccl3*, *F4/80* and *cTNT* at 37°C overnight Following nuclear counterstaining with DAPI. The tissues mentioned above were subjected to observation using a confocal microscope (Zeiss, LSM 780). The primer sequences were shown in Table S6.

*Luciferase reporter assay*: In 24-well plates, HEK 293T cells were co-transfected with either pGL3-*Ccl3* luciferase or its mutant constructs, together with Renilla and pCDH-*Ets2* or control plasmid. After 24 hours, cells were lysed, and luciferase signals were detected by a luminometer (Promega, E1910), and normalized to Renilla control. The primer sequences used for plasmid construction were in Table S7.

*Enzyme-linked immunosorbent assay (ELISA)*: The concentration of POSTN in the mouse serum was determined using the mouse POSTN ELISA kit (R&D Systems) following the manufacturer’s guidelines.

*Propidium iodide staining (PI staining)*: The culture supernatant of cells was harvested in the sample detection tubes. Following PBS washing, cells were digested using 0.25% trypsin and subsequently resuspended in DMEM containing 1% P/S and 10% FBS and then collected in the sample detection tubes. The solution to be tested was incubated with 5 μg/ml Propidium iodide for 10 minutes. Flow cytometry (ACEA Biosciences, Quanteon) was used to analyze the treated cells.

**Supplementary figures**

**Figure S1.** POSTN is significantly elevated in the myocardium and serum and facilitates fibroblast activation. A) Representative immunohistochemical staining of POSTN in cardiac sections from WT (*DSP^f/+^*) and *DSP*-ACM (*CreDSP^f/+^*) mice at 20 days, 1 month, 4 months, and 7 months. Scale bars, 50 µm. B) ELISA analysis of the levels of POSTN in the serum of WT and *DSP*-ACM mice (n=5). C, D) Relative mRNA (C) (n=3) and protein (D) levels of POSTN in cardiac fibroblasts (CFs) derived from WT and *DSP*-ACM mice. E) Representative immunofluorescence staining of Vimentin (red) in the CFs isolated from hearts of WT and *DSP*-ACM mice. Scale bars, 50 µm. F, G) Relative mRNA (F) (n=3) levels of *Acta2* and protein (G) levels of α-SMA in CFs treated with PBS or rmPOSTN. H) Representative immunofluorescence staining of Vimentin (red) in CFs treated with rmPOSTN. Scale bars, 50 µm. Statistical analyses were performed by unpaired two-tailed Student’s *t*-test (b, c, f). Data are mean±SEM. **P* < 0.05, *****P* < 0.0001.

**Figure S2.** POSTN deficiency rescues myocardium loss and dysfunction but does not restore desmosomal structural defects in ACM mice. A) H&E staining in cardiac sections from WT and *DSP*-ACM mice with or without POSTN deficiency. Scale bars, 50 µm. B) Representative ECG signals of T-wave inversion of WT and *DSP*-ACM mice with or without POSTN deficiency. C) Quantitative analysis of left ventricular internal dimension in diastole (LVIDd) and systole (LVIDs), and posterior wall thickness in diastole (LVPWd) and systole (LVPWs) in WT and *DSP*-ACM mice with or without POSTN deficiency (n=4). D) Representative TEM images of cardiac sections from WT and *DSP*-ACM mice with or without POSTN deficiency. Scale bars, 1 µm. E) mRNA levels of *Postn* in CMs and CFs of AAV9-NC and AAV9-*Tcf21*-Cre-treated mice (n=8). F) Western blot analysis of POSTN in CMs and CFs of AAV9-NC and AAV9-*Tcf21*-Cre-treated mice. G) H&E staining in cardiac sections from WT and *DSP*-ACM mice with or without AAV-mediated cardiac fibroblast-targeted deletion of *Postn*. Scale bars, 50 µm. H) Quantitative analysis of left ventricular internal dimension in LVIDd, LVIDs, LVPWd and LVPWs in WT and *DSP*-ACM mice with or without AAV-mediated fibroblast-targeted deletion of *Postn* (n=4). Statistical analyses were performed by one-way ANOVA analysis followed by Tukey’s multiple comparison tests (C, E, H). Data are mean±SEM. ***P* < 0.01, ****P* < 0.001. ns, no significance.

**Figure S3.** POSTN deficiency reduces inflammation in the myocardium of *DSP*-ACM mice. A) Relative mRNA levels of *CD68*, *F4/80*, *Tnf-α*, *Il-6*, *Il-1β*, and *Il-17* in the myocardium of WT and *DSP*-ACM mice with or without POSTN deficiency (n=8). B) Representative immunofluorescence staining of CD68 (red) in cardiac sections from WT and *DSP*-ACM mice with or without POSTN deficiency. Scale bars, 50 µm. C) ELISA analysis of the protein levels of IL-6 in the serum of WT and *DSP*-ACM mice with or without POSTN deficiency (n=6). Statistical analyses were performed by one-way ANOVA analysis followed by Tukey’s multiple comparison tests (a, c). Data are mean±SEM. **P* < 0.05, *****P* < 0.0001. ns, no significance.

**Figure S4.** POSTN deficiency ameliorates cardiac fibrosis in *DSP*-ACM mice. A) Representative Sirius Red staining of cardiac sections from WT and *DSP*-ACM mice with or without POSTN deficiency. Scale bars, 50 µm. B) Representative immunohistochemical staining of Col1a1 in cardiac sections from WT and *DSP*-ACM mice with or without POSTN deficiency. Scale bars, 50 µm. C, D) Representative immunofluorescence staining of Vimentin (red) (C) and α-SMA (green) (D) in cardiac sections from WT and *DSP*-ACM mice with or without POSTN deficiency. Scale bars, 50 µm. E) Heatmaps illustrating the enrichment expression levels of fibrosis-related pathways-related genes in the myocardium of WT and *DSP*-ACM mice with or without POSTN deficiency (n=3). F-I) Relative mRNA levels of *Fn1*, *Ctgf*, *Mmp2* and *Mmp9* in the myocardium of WT and *DSP*-ACM mice with or without POSTN deficiency (n=5). Statistical analyses were performed by one-way ANOVA analysis followed by Tukey’s multiple comparison tests (F-I). Data are mean±SEM. ***P* < 0.01, ****P* < 0.001, *****P* < 0.0001. ns, no significance.

**Figure S5.** POSTN deficiency attenuates cardiomyocyte necroptosis. A) Representative immunofluorescence staining of p-RIP3 (red) in the myocardium of WT and *DSP*-ACM mice with or without POSTN deficiency. Scale bars, 50 µm. B, C) mRNA levels of *Bcl2*, *Bax*, *Gsdmd* and *Gsdme* in the myocardium of WT and *DSP*-ACM mice with or without POSTN deficiency (n=8). D) The activities of lactate dehydrogenase (LDH) in the cultured supernatant of CMs isolated from WT and *DSP*-ACM mice with or without POSTN deficiency (n=6). E) Western blot analysis of the indicated proteins associated with necroptosis in CMs derived from WT and *DSP*-ACM mice with or without POSTN deficiency. F) Western blot analysis of the indicated proteins associated with necroptosis in AC16 cells transfected without or with si*DSP* in the presence or absence of rhPOSTN. G, H) Relative mRNA levels of *DSP* in NMCMs (G) (n=9) and AC16 cells (H) (n=3) transfected with siNC or si*DSPs*. I, J) The activities of LDH in the cultured supernatant from NMCMs (I) and AC16 cells (J), which were transfected with si*DSP* in the presence or absence of TSZ, rmPOSTN or rhPOSTN (n=8). K) Flow cytometry analysis of propidium iodide (PI) staining and the quantification of cell necrosis rate in AC16 cells transfected with si*DSP* in the presence or absence of rhPOSTN (n=3). Statistical analyses were performed by one-way ANOVA analysis followed by Tukey’s multiple comparison tests (B, C, D, G, H, I, J) and unpaired two-tailed Student’s t-test (K). Data are mean±SEM. **P* < 0.05, ***P* < 0.01, *****P* < 0.0001. ns, no significance.

**Figure S6.** POSTN deficiency attenuates cardiomyocyte necroptosis via the integrin-JNK-RIP3-MLKL axis. A) Western blot analysis of p-JNK and JNK in the myocardium of WT and *DSP*-ACM mice with or without AAV-mediated cardiac fibroblast-targeted deletion of *Postn*. B, C) The mRNA (B) (n=8) and protein (C) levels of ITGβ3 and ITGβ5 in NMCMs transfected with si*Itgβ3* and si*Itgβ5*. D) Western blot analysis of p-JNK, JNK, p-RIP3, RIP3, p-MLKL and MLKL in NMCMs transfected with si*DSP* in the presence or absence of rmPOSTN, as well as si*Itgβ3* or si*Itgβ5*. E, F) The mRNA (E) (n=8) and protein (F) levels of ITGβ3 and ITGβ5 in AC16 transfected with si*Itgβ3* or si*Itgβ5*. G) Western blot analysis of p-JNK, JNK, p-RIP3, RIP3, p-MLKL and MLKL in AC16 cells transfected with si*DSP* in the presence or absence of rhPOSTN, as well as si*Itgβ3* or si*Itgβ5*. Statistical analyses were performed by unpaired two-tailed Student’s *t*-test (b, E). Data are mean±SEM. ****P* < 0.001, *****P* < 0.0001. ns, no significance.

**Figure S7.** Cardiomyocytes are the predominant cellular source of CCL3. A) Western blot analysis of CCL3 in the myocardium of WT and *DSP*-ACM mice with or without AAV-mediated cardiac fibroblast-targeted deletion of *Postn*. B, C) The mRNA (B) (n=10) and protein (C) levels of CCL3 in NCMs and CMs isolated from the hearts of *DSP*-ACM mice. D, E) The mRNA (D) (n=9) and protein (E) levels of CCL3 in CFs, macrophages and CMs isolated from the hearts of *DSP*-ACM mice. F) Representative immunofluorescence co-staining of CCL3 (green) with cTNT (cardiomyocyte) (purple), and F4/80 (macrophage) (red) in cardiac sections of *DSP*-ACM mice. Scale bars, 50 µm. G) RNA-FISH staining of *Ccl3* (green), *cTNT* (cardiomyocyte) (red) and *F4/80* (macrophage) (red) in cardiac sections of *DSP*-ACM mice. Scale bars, 50 µm. H, I) Relative mRNA (H) (n=4) and protein (I) levels of CCL3 in AC16 cells transfected with si*DSP* in the presence or absence of rhPOSTN. J) Western blot analysis of CCL3 in NMCMs transfected with si*DSP* in the presence or absence of rmPOSTN, as well as si*Itgβ3* or si*Itgβ5*. K-O) Relative mRNA (n=8) and protein levels of CCL3 in AC16 cells transfected with si*DSP* in the presence or absence of rhPOSTN, as well as αvβ3/αvβ5 integrin neutralizing antibody or si*Itgβ3*/*β5* (K-M), and PD98059 or SP600125 (N, O). Statistical analyses were performed by one-way ANOVA analysis followed by unpaired two-tailed Student’s *t*-test (B, H), Tukey’s multiple comparison tests (D, K, N). Data are mean±SEM. *****P* < 0.0001.

**Figure S8.** POSTN upregulates CCL3 level by the integrin-JNK-ETS2 signaling. A) Relative mRNA levels of *Ets2*, *Atf2*, *c-Jun*, *Smad4*, and *p53* in the myocardium of WT and *DSP*-ACM mice with or without POSTN deficiency (n=6). B) Western blot analysis of ETS2 in the myocardium of WT and *DSP*-ACM mice with or without AAV-mediated cardiac fibroblast-targeted deletion of *Postn* C) Relative mRNA of *Ets2* in CMs derived from WT and *DSP*-ACM mice with or without POSTN deficiency (n=12). D) mRNA levels of *Ets2* in NMCMs and AC16 cells transfected with siNC or si*DSP* in the presence or absence of rmPOSTN or rhPOSTN (n=8). E) Western blot analysis of ETS2 in NMCMs transfected with si*DSP* in the presence or absence of rmPOSTN, as well as si*Itgβ3* or si*Itgβ5*. F-I) Western blot analysis of ETS2 in AC16 cells transfected with si*DSP* in the presence or absence of rhPOSTN, as well as αvβ3/αvβ5 integrin neutralizing antibody (F) or si*Itgβ3*/*β5* (G), PD98059 or SP600125 (H) or Necrostatin-1 (Nec-1) (I). Statistical analyses were performed by one-way ANOVA analysis followed by Tukey’s multiple comparison tests (A, C, D). Data are mean±SEM. **P* < 0.05, ***P* < 0.01, *****P* < 0.0001. ns, no significance.

**Figure S9.** CCL3 promotes cardiac fibroblast activation and POSTN expression by activating the CCR5-NF-κB signaling. A) Relative mRNA levels of *Ccr1* and *Ccr3* in the myocardium of WT and *DSP*-ACM mice with or without POSTN deficiency (n=3). B-D) Relative mRNA levels of *Acta2* (B) and *Postn* (C) (n=6) and Western blot analysis of the indicated proteins (D) in CFs isolated from *DSP^f/+^Postn^+/+^* mice, which were co-cultured with CMs isolated from *CreDSP^f/+^Postn^+/+^* mice in the presence or absence of Maraviroc. E-G) Relative mRNA levels of *Acta2* (E) and *Postn* (F) (n=6) and Western blot analysis of the indicated proteins (G) in CFs isolated from *DSP^f/+^Postn^+/+^* mice, which were co-cultured with CMs isolated from *CreDSP^f/+^Postn^+/+^* mice in the presence or absence of BAY-11-7082. Statistical analyses were performed by one-way ANOVA analysis followed by Tukey’s multiple comparison tests (a-c, e, f). ***P* < 0.01, ****P* < 0.001, *****P* < 0.0001. ns, no significance.

**Figure S10.** POSTN deficiency doesn’t significantly influence cardiac fibrosis and cardiomyocyte necroptosis in 4-month-old ACM mice. A) H&E staining in cardiac sections from WT and *DSP*-ACM mice with or without POSTN deficiency at the ages of 4 and 8 months, respectively. Scale bars, 50 µm. B) mRNA levels of *Nppa* and *Nppb* in the myocardium of WT and *DSP*-ACM mice with or without POSTN deficiency at the ages of 4 and 8 months, respectively (n=6). C) mRNA levels of *Acta2*, *Col1a1*, *Col3a1* and *Tgfβ3* in the myocardium of WT and *DSP*-ACM mice with or without POSTN deficiency at the ages of 4 and 8 months, respectively (n=6). D, E) Masson’s Trichrome staining and immunohistochemical staining of α-SMA in cardiac sections from WT and *DSP*-ACM mice with or without POSTN deficiency at the ages of 4 and 8 months, respectively. Scale bars, 50 µm. F) Western blot analysis of α-SMA in the cardiac tissue lysates of WT and *DSP*-ACM mice with or without POSTN deficiency at the ages of 4 and 8 months, respectively. G) The activities of LDH in the serum isolated from WT and *DSP*-ACM mice with or without POSTN deficiency at the ages of 4 and 8 months, respectively (n=6). H) Western blot analysis of the indicated proteins associated with necroptosis in the myocardium derived from WT and *DSP*-ACM mice with or without POSTN deficiency at the ages of 4 and 8 months, respectively. I) The protein levels of p-JNK, JNK, ETS2 and CCL3 in the myocardium derived from WT and *DSP*-ACM mice with or without POSTN deficiency at the ages of 4 and 8 months, respectively. Statistical analyses were performed by one-way ANOVA analysis followed by Tukey’s multiple comparison tests (B, C, G). Data are mean±SEM. **P* < 0.1, ***P* < 0.01, ****P* < 0.001, *****P* < 0.0001. ns, no significance.

**Figure S11.** The graphic illustration on the role of POSTN-CCL3 loop in *DSP*-ACM progression. Cardiac myofibroblast-derived POSTN promotes cardiomyocyte necroptosis and CCL3 expression through integrin-JNK signaling. CCL3, in turn, activates myofibroblasts via CCR5-NF-κB to enhance POSTN expression, forming a feedforward loop that contributes to fibrosis and arrhythmogenic cardiomyopathy.

**Supplementary tables**

**Table S1. The sequences of siRNAs for *DSP, Itgβ3 and Itgβ5***

| si*DSP*-1#  Mouse | sense: CACCAACAUCGCUCAGAAGTT |
| --- | --- |
|  | antisense: CUUCUGAGCGAUGUUGGUGUU |
| si*DSP*-2#  Mouse | sense: AUACCCCUGUGACAAGAAUTT |
|  | antisense: AUUCUUGUCACAGGGGUAUUU |
| si*DSP*-1#  Human | sense: GAAGCAAUAAACCCAUUAUTT |
|  | antisense: AUAAUGGGUUUAUUGCUUCTT |
| si*DSP*-2#  Human | sense: GGCCAAGAGAAAGAAAUUATT |
|  | antisense: UAAUUUCUUUCUCUUGGCCTT |
| si*Itgβ3*  Mouse | sense: GGAGAGUCCAACAUCUGUA |
|  | antisense: UACAGAUGUUGGACUCUCC |
| si*Itgβ5*  Mouse | sense: GUGUAUUGGUUACAAGUUA |
|  | antisense: UAACUUGUAACCAAUACAC |
| si*Itgβ3*  Human | sense: CCAAGACUCAUAUAGCAUU |
|  | antisense: AAUGCUAUAUGAGUCUUGG |
| si*Itgβ5*  Human | sense: GCAUCCAACCAGAUGGACUAU |
|  | antisense: AUAGUCCAUCUGGUUGGAUGC |

**Table S2. The sequences of negative control for siRNAs**

| siNC  Mouse | sense: UUCUCCGAACGUGUCACGUTT |
| --- | --- |
|  | antisense: ACGUGACACGUUCGGAGAATT |
| siNC  Human | sense: UUCUCCGAACGUGUCACGUTT |
|  | antisense: ACGUGACACGUUCGGAGAATT |

**Table S3. Individual clinical and genetic characteristics of patients**

| **Patient** | **Age (years)** | **Sex** | **LVEF (%)** | **Clinical characteristics** | **Arrhythmia burden** |
| --- | --- | --- | --- | --- | --- |
| **NC-1** | 42 | Male | NA | Healthy | None |
| **NC-2** | 50 | Male | NA | Healthy | None |
| **NC-3** | 29 | Male | NA | Healthy | None |
| **NC-4** | 14 | Male | NA | Healthy | None |
| **NC-5** | 48 | Male | NA | Healthy | None |
| **ACM-1** | 40 | Male | NA | Severe ACM with frequent PVCs, ventricular tachycardia, and cardiogenic syncope | Electrocardiography demonstrated depolarization/repolarization abnormalities, bundle branch reentrant ventricular tachycardia, torsades de pointes, and ventricular tachycardia exhibiting left bundle branch block morphology with positive R waves in leads II, III, and aVF |
| **ACM-2** | 43 | Female | 30 | Enlarged right ventricle, irregular right ventricular free wall, and moderate mitral regurgitation | Sinus rhythm with wandering atrial pacemaker |
| **ACM-3** | 22 | Male | 54 | Subepicardial fatty infiltration involving the right ventricular anterior wall and right ventricular aspect of the interventricular septum, normal left atrial and ventricular chamber size, and heterogeneous left ventricular wall thickness | Sustained ventricular tachycardia and frequent PVCs |
| **ACM-4** | 19 | Male | 55 | Severe tricuspid regurgitation, impaired right ventricular function, and patent foramen ovale | Non-sustained ventricular tachycardia, ventricular escape beats, intraventricular conduction delay, second-degree atrioventricular block, and complete right bundle branch block |
| **ACM-5** | 35 | Female | 40 | Left ventricular systolic dysfunction with mild mitral and tricuspid regurgitation | Atrial flutter, atrial tachycardia, frequent PVCs, non-sustained ventricular tachycardia, and intraventricular conduction delay |

LVEF, left ventricular ejection fraction; ACM, arrhythmogenic cardiomyopathy; PVCs, premature ventricular contractions; NA, not available; NC, normal control.

**Table S4. Key resources table**

| Reagent or Resource | Source | Identifier |
| --- | --- | --- |
| Antibodies | | |
| Mouse monoclonal anti-Periostin | Adipogen | Cat# AG-20B-0033 |
| Rabbit monoclonal anti-GAPDH | Cell Signaling Technology | Cat# 2118S |
| Rabbit monoclonal anti-Vimentin | Abcam | Cat# ab92547 |
| Rabbit monoclonal anti-CD31 | Cell Signaling Technology | Cat# 77699T |
| Rabbit polyclonal anti-Perilipin | Abcam | Cat# ab3526 |
| Rabbit polyclonal anti-α-SMA | Abcam | Cat# ab52218 |
| Rabbit monoclonal anti-Col1a1 | Cell Signaling Technology | Cat# 72026 |
| Rabbit monoclonal anti-Phospho-RIP3 | Cell Signaling Technology | Cat# 91702S |
| Rabbit monoclonal anti-RIP3 | Cell Signaling Technology | Cat# 15828S |
| Rabbit monoclonal anti-Phospho-MLKL | Abcam | Cat# ab196436 |
| Rat monoclonal anti-MLKL | Sigma-Aldrich | Cat# MABC604 |
| Anti-Integrin αvβ3 antibody | Millipore/Merck | Cat# MAB1976Z |
| Anti-Integrin αvβ5 antibody | Millipore/Merck | Cat# MAB1961F |
| Mouse monoclonal anti-Phospho-JNK | Cell Signaling Technology | Cat# 9255S |
| Rabbit polyclonal anti-JNK | Cell Signaling Technology | Cat# 9252S |
| Rabbit polyclonal anti-CCL3 | Thermo Scientific | Cat# PA5-120000 |
| Rabbit polyclonal anti-ETS2 | ABclonal | Cat# A16844 |
| Rabbit monoclonal anti-Intergrin Linked ILK | Abcam | Cat# ab52480 |
| Rabbit monoclonal anti-Phospho-NF-κB p65 | Cell Signaling Technology | Cat# 3033T; |
| Rabbit monoclonal anti-NF-κB p65 | Cell Signaling Technology | Cat# 8242 |
| Mouse monoclonal anti-CD68 | Abcam | Cat# ab955 |
| Rabbit monoclonal anti-ITGβ3 | ABclonal | Cat# A24844 |
| Rabbit monoclonal anti-ITGβ5 | Selleckchem | Cat# F0632 |
| Chemicals, peptides, and recombinant proteins | | |
| Recombinant Mouse Periostin/OSF-2  Protein | R&D Systems | Cat# 2955-F2-050 |
| Recombinant Human Periostin/OSF-2  Protein | R&D Systems | Cat# 3548-F2-050 |
| PD98059 | MedChemExpress | Cat# HY-12028 |
| SP600125 | MedChemExpress | Cat# HY-12041 |
| Necrostatin-1 | MedChemExpress | Cat# HY-15760 |
| Recombinant Mouse CCL3 Protein | MedChemExpress | Cat# HY-P7255 |
| Maraviroc | MedChemExpress | Cat# HY-13004 |
| BAY-11-7082 | MedChemExpress | Cat# HY-13453 |
| Collagenase II | GIBCO | Cat# 17101015 |
| Collagenase IV | GIBCO | Cat# 17104019 |
| 10x RBC lysis buffer | ebioscience | Cat# 00-4300-54 |
| Critical commercial assays | | |
| Mouse Periostin ELISA Kit | R&D Systems | Cat# DY2955 |
| Mouse IL-6 ELISA Kit | R&D Systems | Cat# DY406 |
| Experimental models: Cell lines | | |
| Mouse: CMs (cardiomyocytes) | This paper | N/A |
| Mouse: NCMs (non-cardiomyocytes) | This paper | N/A |
| Mouse: NMCMs (neonatal mouse cardiomyocytes) | This paper | N/A |
| Mouse: NMCFs (neonatal mouse cardiac fibroblasts) | This paper | N/A |
| Human: AC16 | ATCC | N/A |

**Table S5. Sequences of mouse oligonucleotide primers used for qRT-PCR**

| **Genes** | **Primer sequences** |
| --- | --- |
| *Postn* | Forward: CACTACCACTCAGCACTACTC |
|  | Reverse: CTCCAGTCCTCTGCGAAT |
| *Nppa* | Forward: CAACACAGATCTGATGGATTTCA |
|  | Reverse: CCTCATCTTCTACCGGCATC |
| *Nppb* | Forward: GTCAGTCGTTTGGGCTGTAAC |
|  | Reverse: AGACCCAGGCAGAGTCAGAA |
| *Acta2* | Forward: AAGAGCATCCGACACTGCTGAC |
|  | Reverse: AGCACAGCCTGAATAGCCACATAC |
| *Col1a1* | Forward: GCTCCTCTTAGGGGCCACT |
|  | Reverse: CCACGTCTCACCATTGGGG |
| *Col3a1* | Forward: CTGTAACATGGAAACTGGGGAAA |
|  | Reverse: CCATAGCTGAACTGAAAACCACC |
| *Ctgf* | Forward: GGGCCTCTTCTGCGATTTC |
|  | Reverse: ATCCAGGCAAGTGCATTGGTA |
| *Tgf-β3* | Forward: AGTGGCTGTTGAGGAGAGAGT |
|  | Reverse: GTGTGGGTTGTGGTGATCCTT |
| *Fn1* | Forward: ATGTGGACCCCTCCTGATAGT |
|  | Reverse: GCCCAGTGATTTCAGCAAAGG |
| *Ccl3* | Forward: TGTACCATGACACTCTGCAAC |
|  | Reverse: CAACGATGAATTGGCGTGGAA |
| *Ets2* | Forward: CGGCTCAACACCGTCAATGT |
|  | Reverse: AGTCGTGGTCCTTGGGTTTTC |
| *Ccr5* | Forward: TTTTCAAGGGTCAGTTCCGAC |
|  | Reverse: GGAAGACCATCATGTTACCCAC |
| *F4/80* | Forward: TTGTACGTGCAACTCAGGACT |
|  | Reverse: GATCCCAGAGTGTTGATGCAA |
| *CD68* | Forward: TGTCTGATCTTGCTAGGACCG |
|  | Reverse: GAGAGTAACGGCCTTTTTGTGA |
| *Tnf-α* | Forward: GCCAACGGCATGGATCTC |
|  | Reverse: GCAGCCTTGTCCCTTGAAGAG |
| *Il-6* | Forward: GCTAAGGACCAAGACCATCCAAT |
|  | Reverse: GGCATAACGCACTAGGTTTGC |
| *Il-1β* | Forward: TTGGGCCTCAAAGGAAAGAAT |
|  | Reverse: TGGGTATTGCTTGGGATCCA |
| *Il-17* | Forward: TTTAACTCCCTTGGCGCAAAA |
|  | Reverse: CTTTCCCTCCGCATTGACAC |
| *Mmp2* | Forward: CAAGTTCCCCGGCGATGTC |
|  | Reverse: TTCTGGTCAAGGTCACCTGTC |
| *Mmp9* | Forward: GCGTCGTGATCCCCACTTAC |
|  | Reverse: CAGGCCGAATAGGAGCGTC |
| *DSP* | Forward: GAGTTTGGAAGATCTGAAGCTGA |
|  | Reverse: TTTGGCCGTAGCACTTGTCT |
| *c-Jun* | Forward: CTGCGTCTTAGGCTTCTCC |
|  | Reverse: CTCGCCCAAGTTCAACAA |
| *Smad4* | Forward: ACACCAACAAGTAACGATGCC |
|  | Reverse: GCAAAGGTTTCACTTTCCCCA |
| *p53* | Forward: ACCGCCGACCTATCCTTA |
|  | Reverse: GTTTGGGCTTTCCTCCTT |
| *Atf2* | Forward: CCGTTGCTATTCCTGCATCAA |
|  | Reverse: TTGCTTCTGACTGGACTGGTT |
| *Ccr1* | TCCACTCCATGCCAAAAGAC |
|  | GAAGACCAGATCAGAGACAGC |
| *Ccr3* | TCGAGCCCGAACTGTGACT |
|  | CCTCTGGATAGCGAGGACTG |
| *Gapdh* | Forward: AAATCCCATCACCATCTTCC |
|  | Reverse: TCCACCACCCTGTTGCTGTA |
| *S18* | Forward: AACCCGTTGAACCCCATT |
|  | Reverse: CCATCCAATCGGTAGTAGCG |
| *Itgβ3* | Forward: TGTTTACCACGGATGCCAAGA |
|  | Reverse: ACGCAGTCCCCACAGTTACATT |
| *Itgβ5* | Forward: GCTTAGGTTTCGGGTCTTTTGTT |
|  | Reverse:GCAGAGCATCTTTTCGCCAT |

**Table S6. Primer sequences for FISH**

| **FISH probe** | **Sequence (5’-3’)** |
| --- | --- |
| FAM-*Postn* Mouse | GCGTTGGTCCATGCTCAGAGTGTC |
| TAMRA-*Vimentin* Mouse | GCAAGGATTCCACTTTCCGTTCA |
| FAM-*Ccl3* Mouse | tctgccggtttctcttagtcaggaaaatgacacctggctgggagca |
| FAM-*Ccl3* Human | ctgccggcttcgcttggttaggaagatgacaccgggcttggagca |
| TAMRA-*F4/80* Mouse | atggccttgaaggtcagcaacctcgtgtccttgagtttagagact |
| TAMRA-*F4/80* Human | Acctcttgtattcttctcgtacctggccgttgagcagacagt |
| TAMRA-*cTNT* Mouse | Acagctccttggccttctctctcagttggtcttcattcaggt |
| TAMRA-*cTNT* Human | Cctgtttcggagaacattgatctcatatttctgctgcttgaact |

**Table S7. Primers of plasmids for luciferase reporting assay**

| **Genes** | **Vector** | **Primer sequences** |
| --- | --- | --- |
| *Ccl3* promotor Human | pGL3-basic | Forward: GTCGCACTTGGCTTCTGTC |
|  |  | Reverse: AGGACCCTAGCTTAGTATGGATTT |
| *Ets2*  Human | pCDH | Forward: ATGAATGATTTCGGAATCAA |
|  |  | Reverse: TCAGTCCTCCGTGTCGGGCT |
